# Host Plasma Microenvironment in Immunometabolically Impaired HIV Infection Leads to Dysregulated Monocyte Function and Synaptic Transmission *Ex Vivo*

**DOI:** 10.1002/advs.202416453

**Published:** 2025-02-27

**Authors:** Flora Mikaeloff, Marco Gelpi, Alejandra Escós, Tianqi Wang, Soham Gupta, Anna Olofsson, Sara Svensson Akusjärvi, Sabrina Schuster, Prajakta Naval, Vikas Sood, Negin Nikouyan, Andreas D. Knudsen, Beate Vestad, Julie Høgh, Johannes R. Hov, Thomas Benfield, Marius Trøseid, Vinay Pawar, Marijana Rucevic, Rui Benfeitas, Ákos Végvári, Liam O'Mahony, Rajkumar Savai, Niklas K. Björkström, Magda Lourda, João Pedro de Magalhães, Siegfried Weiss, Adil Mardinoglu, Mukesh Kumar Varshney, Annika C. Karlsson, Yasir Ahmed Syed, Susanne D. Nielsen, Ujjwal Neogi

**Affiliations:** ^1^ The Systems Virology Lab, Division of Clinical Microbiology, Department of Laboratory Medicine Karolinska Institutet Huddinge 141 52 Sweden; ^2^ Copenhagen University Hospital Rigshospitalet Copenhagen 2100 Denmark; ^3^ Neuroscience and Mental Health Innovation Institute and School of Biosciences, Hadyn Ellis Building Cardiff University Cardiff CF24 4HQ UK; ^4^ Division of Clinical Microbiology, Department of Laboratory Medicine Karolinska Institutet Huddinge 141 52 Sweden; ^5^ Division of Chemistry I, Department of Medical Biochemistry and Biophysics Karolinska Institutet Solna 171 65 Sweden; ^6^ Research Institute of Internal Medicine Oslo University Hospital Rikshospitalet Oslo 0372 Norway; ^7^ Norwegian PSC Research Center Oslo University Hospital Rikshospitalet Oslo 0372 Norway; ^8^ Institute of Clinical Medicine University of Oslo Oslo 0313 Norway; ^9^ Department of Infectious Diseases Copenhagen University Hospital – Amager and Hvidovre Hvidovre 2650 Denmark; ^10^ Institute of Clinical Medicine Oslo 0372 Norway; ^11^ Olink AB Uppsala 753 30 Sweden; ^12^ National Bioinformatics Infrastructure Sweden (NBIS), Science for Life Laboratory, Department of Biochemistry and Biophysics Stockholm University Solna 171 65 Sweden; ^13^ Departments of Medicine and Microbiology, APC Microbiome Ireland University College Cork Cork T12 K8AF Ireland; ^14^ Lung Microenvironmental Niche in Cancerogenesis, Institute for Lung Health (ILH) Justus Liebig University 35392 Giessen Germany; ^15^ Department of Lung Development and Remodeling, Max Planck Institute for Heart and Lung Research, member of the German Center for Lung Research (DZL) Member of the Cardio‐Pulmonary Institute (CPI) 61231 Bad Nauheim Germany; ^16^ Center for Infectious Medicine, Department of Medicine Huddinge, Karolinska Institutet Karolinska University Hospital Huddinge 141 52 Sweden; ^17^ Childhood Cancer Research Unit Department of Women's and Children's Health Karolinska Institutet Solna 171 77 Sweden; ^18^ Genomics of Ageing and Rejuvenation Lab Institute of Inflammation and Ageing University of Birmingham Birmingham B15 2WB UK; ^19^ Department of Molecular Immunology Helmholtz Centre for Infection Research 38124 Braunschweig Germany; ^20^ Science for Life Laboratory KTH – Royal Institute of Technology Solna 171 65 Sweden; ^21^ Centre for Host‐Microbiome Interactions Faculty of Dentistry Oral & Craniofacial Sciences King's College London London GW37+C7 UK

**Keywords:** HIV/AIDS, Integrative omics, patient stratification, personalized metabolic models

## Abstract

Risk stratification using multi‐omics data deepens understanding of immunometabolism in successfully treated people with HIV (PWH) is inadequately explained. A personalized medicine approach integrating blood cell transcriptomics, plasma proteomics, and metabolomics is employed to identify the mechanisms of immunometabolic complications in prolonged treated PWH from the COCOMO cohort. Among the PWHs, 44% of PWH are at risk of experiencing immunometabolic complications identified using the network‐based patient stratification method. Utilizing advanced machine learning techniques and a Bayesian classifier, five plasma protein biomarkers; Tubulin Folding Cofactor B (TBCB), Gamma‐Glutamylcyclotransferase (GGCT), Taxilin Alpha (TXLNA), Pyridoxal Phosphate Binding Protein (PLPBP) and Large Tumor Suppressor Kinase 1 (LATS1) are identified as highly differentially abundant between healthy control (HC)‐like and immunometabolically at‐risk PWHs (all FDR<10^−10^). The personalized metabolic models predict metabolic perturbations, revealing disruptions in central carbon metabolic fluxes and host tryptophan metabolism in at‐risk phenotype. Functional assays in primary cells and cortical forebrain organoids (FBOs) further validate this. Metabolic perturbations lead to persistent monocyte activation, thereby impairing their functions *ex vivo*. Furthermore, the chronic inflammatory plasma microenvironment contributes to synaptic dysregulation in FBOs. The endogenous plasma inflammatory microenvironment is responsible for chronic inflammation in treated immunometabolically complicated at‐risk PWH who have a higher risk of cardiovascular and neuropsychiatric disorders.

## Graphical abstract

Network‐based risk stratification of people living with HIV (PWH) on prolonged therapy using multi‐omics data identifies 44% of the patients are at risk of immunometabolic complications. A senescence‐associated myeloid cell‐driven plasma microenvironment drives immunometabolic dysregulation in at‐risk PWH. Ex vivo organoid assays demonstrate disrupted neuronal network properties and synaptic protein expression due to metabolically linked inflammatory microenvironment.

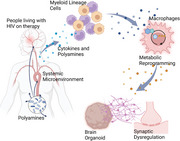

## 1 Introduction

The complex and multifactorial relationship between immune and metabolic function in individuals living with HIV (PWH) receiving antiretroviral therapy (ART) can provide additional insights beyond clinical phenotypes for PWH.^[^ ^1^ ^]^ While ART can help to control HIV replication and improve immune function, prolonged ART can also contribute to metabolic dysregulation and chronic inflammation.^[^ ^2^ ^]^ Early‐generation ART can contribute to mitochondrial dysfunction,^[^ ^2b^ ^]^ impacting immune and metabolic function. A fraction of the PWH receiving long‐term ART experience clinical complications, including chronic comorbidities, mitochondrial toxicities after early‐generation ART, metabolic abnormalities, and adipose tissue redistribution.^[^ ^3^ ^]^

The systems‐level analyses of integrative multi‐omics can represent a functional readout of the biological pathways, which is not convoluted in understanding the pathobiology of PWH with long‐term ART. Earlier global or cell‐type specific blood transcriptomic studies have provided valuable insights into biological mechanisms of disease progression, susceptibility to infection, or natural immune control mechanisms.^[^ ^2^, ^4^ ^]^ No studies have been performed to identify personalized or disease‐specific immune signatures after prolonged successful ART in PWH. These could provide novel mechanistic insights that explain complex relationships and systematic understanding of the disease phenotype and immune status in PWH. Our recent Copenhagen Comorbidity Cohort (COCOMO) systems biology study identified PWH with at‐risk metabolic complications by applying molecular data‐driven network‐based patient stratification despite a favorable HIV‐related clinical profile.^[^ ^1^ ^]^ However, the molecular mechanism underlying the dysregulated metabolic traits in PWH is not yet fully elucidated.

The main objective of this study was to identify the mechanisms of immunometabolic complications, i.e., dysregulation or disruption in the balance between immune function and metabolism in prolonged treated PWH through personalized multi‐omics data‐driven health profiling. Here, we performed genome‐wide transcriptomics analysis of the peripheral blood mononuclear cells (PBMCs) and high‐throughput plasma proteomics analysis targeting nearly 3000 proteins in a cohort of 158 HIV‐infected individuals with prolonged successful treatment. We applied network‐based patient stratification of PWH at risk of clinical complications and identified the plasma protein biomarkers of the at‐risk phenotype by using advanced machine learning algorithms. Further, we implemented disease state‐specific and personalized metabolic models to predict the biological mechanism of the metabolic perturbation behind the at‐risk phenotype. Finally, we validated the findings using functional assays in primary cells and complex human induced pluripotent stem cells (iPSCs) differentiated functional cortical forebrain organoids (FBOs). Identifying the mechanism behind the clinical complications despite successful immune reconstitution and viral suppression opens new avenues for metabolic perturbation targeted therapy for improving the health of PWH.

## 2 Results

### 2.1 Multi‐Omics‐Driven Network Analysis Identifies PWH with Clinical Complications

**Figure 1:**
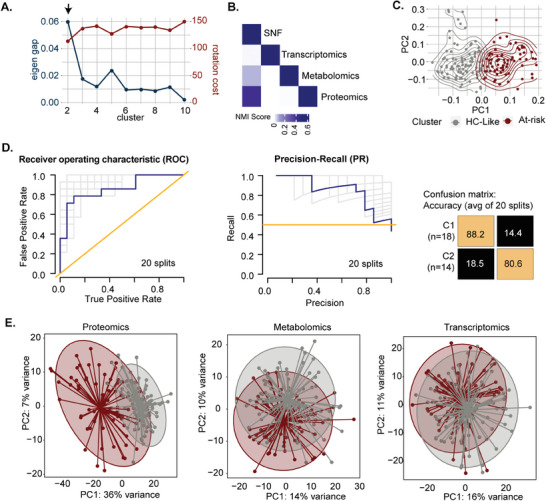
Similarity network fusion‐based PWH stratification. A) Eigengap and rotation cost per number of clusters in SNF network. The cluster number is indicated by an arrow where maximal Eigen gap = 0.06 and minimum rotation cost = 117. B) Concordance matrix of NMIs between the fused network and every single omic network. Values in NMI (data set) are 0.4 (proteomics), 0.07 (metabolomics), and 0.008 (transcriptomics). C) The PCA of the fused network is segregated based on the cluster. Sample color is based on cluster. D) NetDx performance results in predicting SNF risk clusters based on omics data and clinical parameters. Receiver operating characteristic (ROC) curve (left), precision‐recall (PR) curve (middle), and an average of confusion matrices (right) for 20 data splits train/test. E) PCA of the individual omics to identify the heterogeneity of the clusters based on the single omic.

The patient populations (*n* = 158) included in this study were selected randomly and were part of the larger COCOMO cohort (*n* = 1099) of PWH with successful ART. We also used 155 clinical, demographic, and lifestyle parameters from the COCOMO database (Table S1, Supporting Information).^[^ ^1^ ^]^ The key clinical features resembled the overall cohort at the time of sample collection (Table S2, Supporting Information). We performed the PBMC RNAseq data (Illumina NovaSeq6000) and plasma secretome (Olink^®^ Explorer 3072) and used untargeted plasma metabolomics from our earlier study^[^ ^5^ ^]^ to stratify the PWH based on molecular data. Two clusters of PWH were identified based on two heuristics from the similarity network fusion (SNF) of the transcriptomics, proteomics, and metabolomics (**Figure** **1**A). The clustering was driven mainly by proteomics (Figure 1B) and showed good separation of samples based on SNF (Figure 1C). The patient similarity network further validated the SNF classification with high precision (mean accuracy = 0.84) (Figure 1D).

When the single omics layers were analyzed, the proteomics dataset demonstrated the highest proportion of variance explained by the first two principal components (PC1: 36%, PC2: 7%) compared to metabolomics (PC1: 14%, PC2: 10%) and transcriptomics (PC1: 16%, PC2: 11%). This further supports that proteomics captures more dominant patterns in the data, as observed in the SNF (Figure 1E). Clinically, we defined the two groups as healthy control‐like (HC‐like herein) PWH with a median (IQR) CD4/CD8 ratio of 1.05 (0.68‐1.33) and at‐risk of immunometabolic complications (at‐risk herein) with a median (IQR) CD4/CD8 ratio of 0.78 (0.55‐1.06) (*p* < 0.05). The at‐risk group displayed increased visceral adipose tissue (VAT), subcutaneous adipose tissue (SAT), hypertension, waist circumference, and systolic blood pressure (all *p* < 0.05) compared to the HC‐like group (**Table** **1** and Table S1, Supporting Information). Based on the coronary artery disease (CAD) data, we categorized it into no atherosclerosis or normal (no stenosis), non‐obstructive CAD (atherosclerosis that obstructs 1%–49% of the lumen), and obstructive CAD (atherosclerosis that obstructs 50% or more of the lumen) groups. Though there were no statistically significant differences (*p* = 0.158), the at‐risk group had a higher incidence of non‐obstructive CAD (39% vs. 28%) and obstructive CAD (20% vs. 15%) than the HC‐like group (Table 1). This further emphasizes our earlier conclusions that relying solely on clinical data cannot offer comprehensive insights into the intricate dysregulated metabolic traits observed in PWH.^[^ ^1^ ^]^

**Table 1:** Patient demographical, HIV‐related, clinical, and lifestyle data. Only significant and relevant clinical and lifestyle data are presented.

	SNF‐1 [HC‐like]	SNF‐2 [At‐risk]	*P* value
N	88	70	
Age in years, Median (IQR)	52 (47–61.25)	54 (47–63)	0.13^Ø)^
Sex, Male, N (%)	73 (83)	66 (94)	0.053^*)^
Ethnicity Caucasian, N (%)	70 (80)	66 (97)	0.003^*)^
Mode of transmission, N (%) Homosexual/bisexual Heterosexual Other/unknown	57 (65) 23 (26) 8 (9)	53 (77) 12 (17) 4 (6)	0.26^*)^
CD4 Nadir, cells mL^−1^, Median (IQR)	229 (100–300)	231.5 (91.5–300)	0.41^Ø)^
CD4 at ART Initiation, cells mL^−1^, Median (IQR)	240 (142.5–350)	279 (82–320)	0.84^#)^
Viral Load at ART initiation, log copies mL^−1^, Median (IQR)	5.08 (4.3–5.7)	4.94 (4.31–5.34)	0.51^#)^
CD4 at sampling, cells mL^−1^, Median (IQR)	685 (558–933)	691 (530–828)	0.206^Ø)^
CD8 at sampling, cells mL^−1^, Median (IQR)	740 (575–950)	835 (641–1270)	0.053^#)^
CD4/CD8 ratio, Median (IQR)	1.05 (0.68–1.33)	0.78 (0.55–1.06)	0.007^#)^
Viral load (<50 copies mL^−1^), N (%)	86 (99)	67 (96)	0.32^**)^
Duration of treatment in years, median (IQR)	15 (8‐18)	15 (6–19)	0.81^#)^
Current Treatment, 1st drug, N (%) ABC TDF/TAF Other	39 (44) 45 (51) 4 (5)	21 (30) 43 (61) 6 (9)	0.17^**)^
Current Treatment, 3rd drug, N (%) NNRTI PI/r INSTI	45 (51) 24 (27) 19 (22)	29 (43) 19 (28) 20 (29)	0.46^*)^
Previous exposure to DDI/d4T/AZT, N (%)	62 (70)	46 (66)	0.64^*)^
BMI, kg/m2, Median (IQR)	24.45 (21.98‐27.3)	25.25 (22.77–27.82)	0.40^#)^
VAT, Median (IQR)	79.8 (37.3‐124.1)	123.55 (95.15–177.95)	< 0.001^#)^
SAT, Median (IQR)	125.6 (78‐184.4)	113.05 (80.6–179.32)	0.61^#)^
Hypertension, N (%)	35 (40)	43 (61)	0.01^*)^
Central obesity, N (%)	50 (57)	51 (73)	0.055^*)^
Beef intake (times per week)	2 (1‐2)	1 (1‐2)	0.04^#)^
Fruit intake < 1 per month > 1 per week >1 per day	12(13.95%) 36(41.86%) 38(44.19%)	1(1.43%) 29(41.43%) 40(57.14%)	0.014^*)^
Cold Fish intake (times per week)	2(1‐5)	3(2–7)	0.014^#)^
Smoking status Never smoked Ex‐Smoker Current smoker	44(50%) 24(27.27%) 20(22.73%)	25(35.71%) 33(47.14%) 12(17.14%)	0.03^*)^
Waist circumference (cm)	94 (86‐102.25)	99.5 (91.25–105)	0.02^#)^
Systolic blood pressure (right arm, mm mercury)	128 (121‐139.25)	137.5 (127.75–145)	0.002^#)^
Alaninaminotransferas, IU L^−1^, Median (IQR)	22.5 (17‐31.25)	30.5 (22–39.75)	0.0024^Ø)^
eGFR, reads mL min^−1^/1.73m2, Median (IQR)	88.86 (78.78‐98.07)	86.8 (75.84–92.52)	0.054^#)^
Triglyceride, mmol L^−1^, Median (IQR)	1.71 (1.21‐2.6)	2.14 (1.41–3.26)	0.034^Ø)^
High‐density lipoprotein, mmol L^−1^, Median (IQR)	1.21 (0.92‐1.51)	1.04 (0.78–1.35)	0.03^Ø)^
Coronary Artery Disease (CAD), n (%) Normal Non‐obstructive CAD Obstructive CAD	50 (57%) 25 (28%) 13 (15%)	29 (41%) 27 (39%) 14 (20%)	0.158^*^ ^)^

^Ø)^ Student's T‐test;

^#)^ Mann Withney U test;

*) Chi‐square test;

**) Fisher exact test.

### 2.2 Plasma Biomarkers Determine the At‐Risk Phenotype Associated with Myeloid Cell Senescence

**Figure 2:**
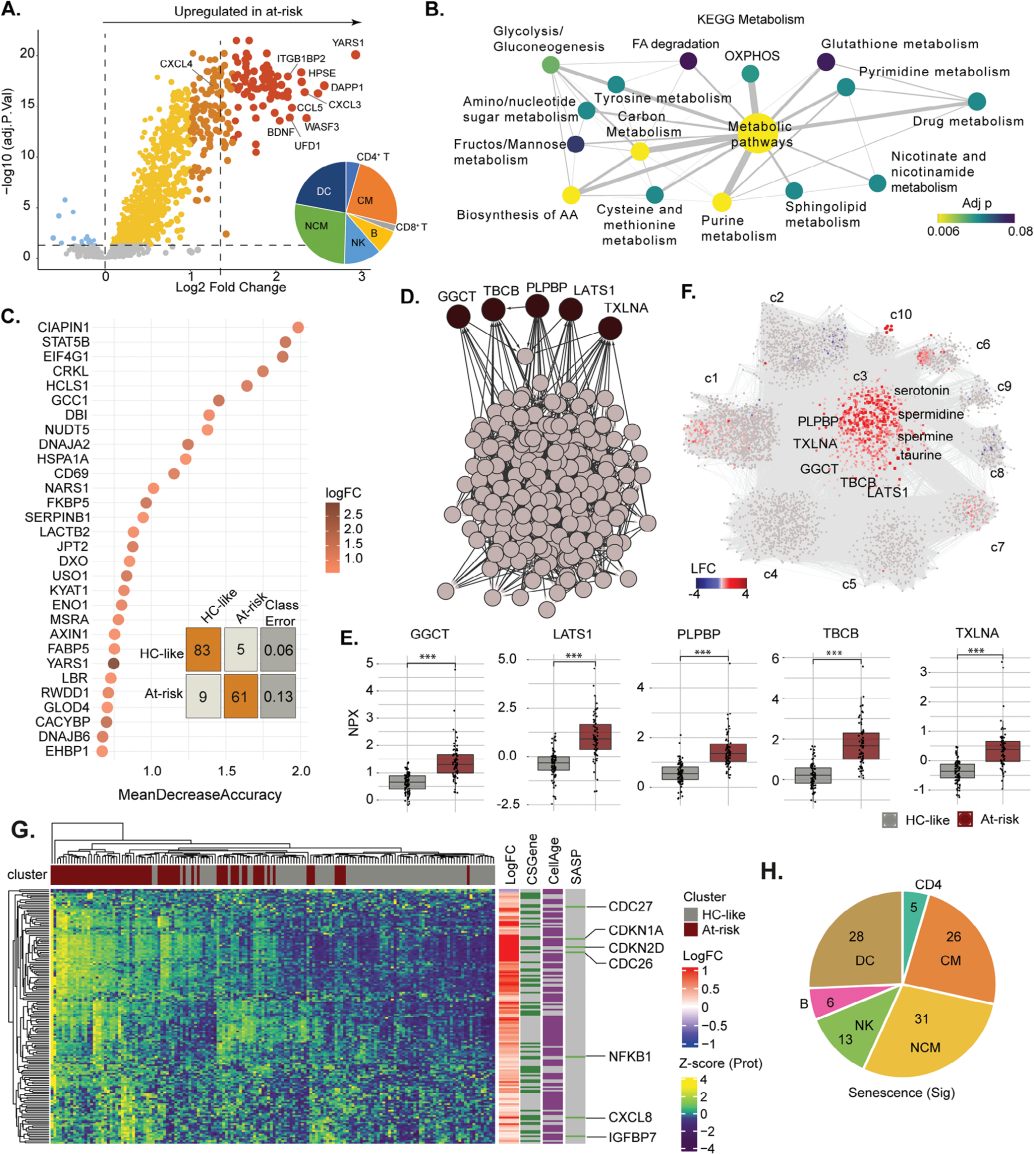
Identification of key molecules and pathways based on proteomics analysis. A) Volcano plot based on proteomics data analysis representing differential abundance between HC‐like and at‐risk clusters. Up‐regulated proteins are shown in red, while down‐regulated proteins are represented in blue. Inset pie chart representing the number of proteins per cell type differentially expressed compared to the other cell types in single‐cell transcriptomics data. B) The Cytoscape network was based on identifying over‐represented KEGG pathways associated with the metabolism based on proteins differing from at‐risk clusters. The node size is proportional to the number of proteins annotated with this pathway and color varies based on p‐value. C) Importance plot based on mean decrease accuracy from random forest model for the prediction of clusters based on proteomics data (Boruta, iterations = 1000, features = 1000). A confusion matrix is also displayed. D) Directed acyclic graph based on 187 most important proteins differing proteomics data at‐risk and HC‐like patients and top 5 proteins with the highest degree of influence (top of the graph) are labelled. E) Boxplots of driver proteins separated by groups. Color is based on cluster and condition (HC‐like = grey, at‐risk = red). *P* values are displayed for each comparison (LIMMA, FDR < 0.1 (*), FDR < 0.1 (**), FDR < 0.1 (***)). F) Co‐expression network after communities' detection based on transcriptomics, metabolomics, and proteomics. Features differing between HC‐like and at‐risk patients are colored red if upregulated or blue if downregulated. Driver genes identified by structural causal modeling are labeled. G) Heatmap based on senescence‐associated protein markers differing between HC‐like and at‐risk clusters of patients. The patient cluster is indicated above, and protein Fold Change and annotation to senescence databases are on the right. Data were Z‐score transformed. H) Pie chart representing the number of senescence‐associated proteins per cell type differentially expressed compared to the other cell types in single‐cell transcriptomics data.

The patient stratification identified that proteomics had the highest impact on the clustering (Figure 1B). Therefore, we aimed to identify the biological mechanisms of systemic immunometabolic dysregulation in at‐risk PWH, analyzing proteomics data alone. The differential protein abundance (DPA), after adjustment for ethnicity, smoking, fruit, and beef intake, identified 1141 proteins differing between the groups (False Discovery Rate; FDR<0.05), of which 1120 proteins were highly abundant in the at‐risk PWH (**Figure** **2**A and Table S3, Supporting Information). Based on the expression of the genes in single‐cell RNA sequencing (scRNAseq) data [(*n* = 11 860 cells) downloaded from 10X Genomics from healthy PBMCs], the significantly altered proteins were mainly expressed by the myeloid lineage cells [classical monocytes (CM), nonclassical monocytes (NCM), and dendritic cells] (Figure 2A inset). To systematically identify enriched categories of proteins and the associated global molecular pathways, we performed Gene Ontology (GO) enrichment analysis using BiNGO v3.0.3 (Table S4, Supporting Information). The top 30 over‐represented GO categories were mainly associated with the metabolic and cellular processes linked to response to external factors like stress that potentially regulate cell death and proliferation (Figure S1, Supporting Information). Therefore, we used directed protein set enrichment analysis using the Kyoto Encyclopedia of Genes and Genomes (KEGG) restricted to metabolic pathways to identify the specific metabolic process. We identified the amino acid (AA) linked modulation of the central carbon metabolism (FDR<0.1) (Figure 2B and Table S5, Supporting Information). As AA‐metabolism significantly influences metabolic rewiring and facilitates diverse immune cell functions,^[^ ^6^ ^]^ we posit that the altered AA‐metabolism caused immunometabolic complications in the at‐risk PWH. Therefore, we aimed to identify biomarkers defining the at‐risk PWH. In a stepwise manner, we first use the Random Forest (RF) consensus feature selection to reduce the number of features, followed by Bayesian Belief Networks (BBNs) to narrow down the list of biomarkers. The RF consensus feature selection identified 187 proteins that separated the clusters (Accuracy = 91.14, Sensitivity = 0.94, Specificity = 0.87) (Figure 2C). We used the structural causal modeling (SCM) method to improve further the biomarker prediction based on these 187 most informative proteins for cluster separation identified in RF. The directed acyclic graph (DAG) represented the causal effects of proteins among each other, and five driver proteins were identified to have a decisive influence on the whole network structure (Figure 2D). Tubulin Folding Cofactor B (TBCB) had the most influence on the network [Bayesian Information Criterion (BIC) difference = 25.6], followed by Gamma‐Glutamylcyclotransferase (GGCT) (DBIC = 12.57), Taxilin Alpha (TXLNA) (DBIC = 8.6), Pyridoxal Phosphate Binding Protein (PLPBP) and Large Tumor Suppressor Kinase 1 (LATS1). These proteins identified were also highly differentially abundant between HC‐like and at‐risk (all FDR<10^−10^) (Figure 2E). Given that five proteins were identified in the BBNs, we further aimed to identify how these proteins influenced the systemic dysregulation in the at‐risk PWH by performing a multi‐omics weighted co‐expression analysis. The consensus association network consisted of 4536 nodes and 381 404 edges belonging to ten communities identified by the Leiden algorithm. The most central community (c3) (centrality = 36, *n* = 705) was mainly driven by proteins (*n* = 683) and metabolites (*n* = 21) (Figure 2F; Table S6, and Figure S2, Supporting Information) and contained all the driver proteins. Interestingly, 81% (85/105) of the significant senescence‐associated proteins (SAP) [defined by combining the detected proteins Senescence‐Associated Secretory Phenotype (SASP), cell senescence genes database, and the CellAge database] differing between HC‐like and at‐risk (FDR<0.05) were part of the central community of the protein network indicative the role of the driver proteins in the senescence process in the at‐risk PWH (Figure S2, Supporting Information). The key metabolites of the central community were serotonin, taurine, spermine (SPR), and spermidine (SPD), which are influenced by the gut microbiota. To identify whether the SAP retain the cluster property, we performed the hierarchical clustering analysis (HCA) restricting to significantly different SAP (*n* = 151, FDR<0.05). We identified the retention of the cluster property (Figure 2G). Among them, seven SASP proteins had a higher abundance in at‐risk compared to HC‐like, indicating an accumulation of senescent features within the at‐risk group (Figure S3, Supporting Information). The significant senescent markers that differed between the HC‐like and at‐risk PWHs were mainly expressed by the myeloid lineage cells (Figure 2H). Based on our assertion regarding the altered amino acid metabolism causing immunometabolic complications in at‐risk PWH, our data further reveal that the senescence‐associated myeloid cell lineage‐driven plasma microenvironment contributes to the rationale behind this immunometabolic dysregulation in at‐risk PWH.

### 2.3 Microenvironment Orchestrates the Monocyte and Monocyte‐Derived Macrophage Function *Ex Vivo*

**Figure 3:**
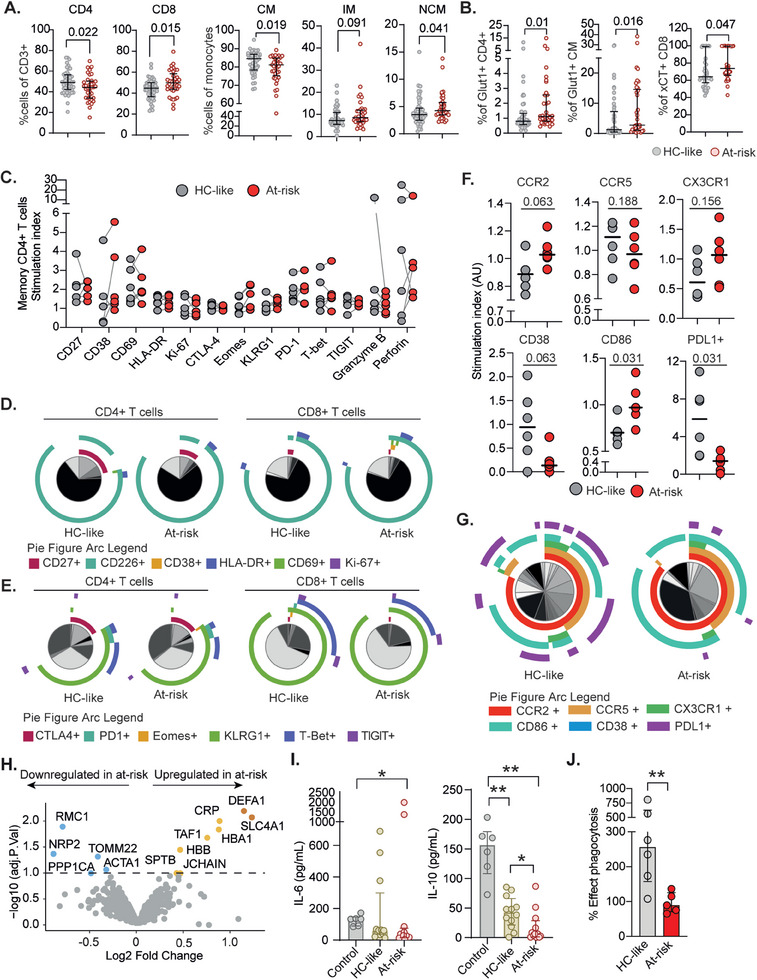
Flow cytometry T‐cells and monocytes A) Dot plot showing the CD4^+^ and CD8^+^ T‐cells proportion of the CD3^+^ T‐cells and classical (CM), intermediate (IM), and non‐classical monocytes (NCM). *P* values are presented. B) Percentage of Glut1 positive cells on CD4+ T‐cells and CM and cystine/glutamate antiporter xCT positive cells on CD8 cells that were significant (*p *< 0.05) None of the other receptors were statistically significant. C) Dot plot showing the expression of phenotypic and functional markers on memory CD4+ T cells stimulated in the presence of either HC‐like or at‐risk plasma. The stimulation index represents the fold change relative to the negative control. Only markers with modest expression across donors are shown. Gray lines connect paired samples. Friedman test. D) Pie charts depict activation markers' co‐expression on memory CD4+ and CD8+ T cells after incubation with HC‐like or at‐risk plasma. Permutation test. E) Pie charts depict co‐inhibitory and transcription factor marker co‐expression on memory CD4+ and CD8+ T cells after incubation with HC‐like or at‐risk plasma. Permutation test. F) Dot plot showing the expression of chemokine receptors (CCR2, CCR5, and CX3CR1), activation markers (CD38 and CD86), and expression of PDL1+ on monocytes after incubation with HC‐like (*n* = 10) or at‐risk (*n* = 10) plasma for 48 h with LPS stimulation (1 pg ml^−1^). The stimulation index represents the fold change relative to the negative control. G) Pie charts depict activation and co‐inhibitory markers' co‐expression of monocyte markers. H) Volcano plots show the differing proteins between monocytes treated with the pool of non‐homologous plasma from at‐risk (*n* = 10) PWH versus those treated with a pool of HC‐like (*n* = 10) PWH plasma for 48 h with LPS stimulation (1 pg ml^−1^). I) Measurement of IL‐6 and IL‐10 in the supernatant following differentiation of the MDM for seven days with GM‐CSF from HIV‐negative controls (*n* = 6), HC‐like (*n* = 12), and at‐risk (*n* = 12) PWH. J) Phagocytic functions were determined in MDM from HC‐like (*n* = 6) and at‐risk (*n* = 5) PWH.

Based on the above findings, we hypothesized that the plasma microenvironment affects the function of monocytes when depleted by prolonged activation or chronic inflammation.^[^ ^7^ ^]^ To investigate this, we first measured the proportion of CD4^+^ and CD8+ T‐cells and classical (CM), intermediate (IM), and nonclassical (NCM) monocytes in HC‐like (*n* = 48) and at‐risk (*n* = 37) PBMCs as described by us recently.^[^ ^8^ ^]^ The CD4+ T‐cells were decreased (*p* = 0.022) while CD8^+^ T‐cells (*p* = 0.015) were increased in at‐risk PWH compared to HC‐like PWH supporting the clinical observations (**Figure** **3**A). Interestingly, there was a decrease in CM (*p* = 0.019) and increase in NCM (*p* = 0.041) in at‐risk PWH potentially due to the inflammatory conditions (Figure 3A). As the majority of the proteins dysregulated were part of the metabolic process (Figure 2B), we further analyzed the metabolite transporters, glucose transporter‐1 (Glut1), pyruvate and lactate transporter monocarboxylate transporter 1 (MCT‐1), and cysteine/glutamate antiporter (xCT). The receptor expression analysis showed that the percentage of Glut1^+^ CD4^+^ T‐cells and CM and xCT^+^ CD8^+^ T‐cells were increased in at‐risk PWH compared to HC‐like. The increase in metabolite receptor expression might be linked to alterations in immunometabolism, impacting the functional properties of these immune cells. To investigate this, we treated donor PBMCs (*n* = 6) as a co‐culture system with non‐homologous pooled plasma from HC‐like (*n* = 10) and at‐risk (*n* = 10) PWH. We stimulated the PBMCs with either lipopolysaccharide for monocyte polarization or pooled viral peptides (CEF, cytomegalovirus, Epstein‐Barr virus, and flu virus) for CD4^+^ and CD8^+^ T cell activation and exhaustion. There was no significant difference in the expression of phenotypic, inhibitory checkpoints, transcriptional, or functional markers on memory CD4^+^ or CD8^+^ T‐cells exposed to HC‐like and at‐risk plasma (Figure 3C; Figure S4A, Supporting Information). Likewise, no differences were observed in the polyfunctional profiles of the memory CD4^+^ or CD8^+^ T‐cells (Figure 3D,E; Figure S4B, Supporting Information). However, in the case of the monocyte functionality assay (Figure S4C, Supporting Information), there was a significantly increased expression of CD86 (*p* = 0.031) and a trend for the C‐C chemokine receptor type 2 (CCR2) (*p* = 0.063) and C‐X3‐C motif chemokine receptor 1 (CX3CR1) (*p* = 0.156) in at‐risk plasma‐treated monocytes compared to the HC‐like plasma treatment (Figure 3F). On the contrary, the expressions of CD38 (*p* = 0.063) and PDL1 (*p* = 0.031) in the at‐risk plasma‐treated cells were lower than in the HC‐like plasma treatment. The polyfunctionality of the monocytes recapitulated the same (Figure 3G). This data suggests that the inflammatory microenvironment led dysfunctional monocytes to at‐risk phenotype. Further, we treated the isolated monocytes from six donors with non‐homologous plasma from HC‐like and at‐risk PWH. The quantitative proteomics identified upregulation of C‐reactive protein (CRP) and defensin 1 (DEFA1) indicative of activation of inflammatory response. On the contrary, proteins like neuropilin 2 (NRP2) that regulate monocyte migration, activation, and differentiation were downregulated. The translocase of outer mitochondrial membrane 22 (TOMM22) was downregulated (Figure 3H and Table S7, Supporting Information). We further differentiated the monocytes from HIV‐negative controls (*n* = 6), HC‐like (*n* = 12), and at‐risk PWH (*n* = 12) and polarized them with GM‐CSF to obtain monocyte‐derived macrophages (MDM). We measured IL‐6 and IL‐10 secretion and observed that the IL‐6 secretion was significantly low in at‐risk compared to control (Figure 3I). Though heterogeneous, there was a suppression of the IL‐10 production in at‐risk plasma‐treated monocytes compared to HC‐like, which could indicate a compromised immune response, leading to reduced anti‐inflammatory and immunoregulatory effects (Figure 3G). Further, we performed the phagocytosis assay on the 7‐day polarized MDM and observed compromised phagocytosis in the at‐risk group (Figure 3J). Overall, this data indicated the critical role of the secretory microenvironment in orchestrating the function and behavior of monocytes and MDM *ex vivo*, with impaired monocyte/macrophage in at‐risk PWH.

### 2.4 Metabolic Modeling Highlights Dysregulation in At‐Risk PWH caused by Tryptophan Metabolism

**Figure 4:**
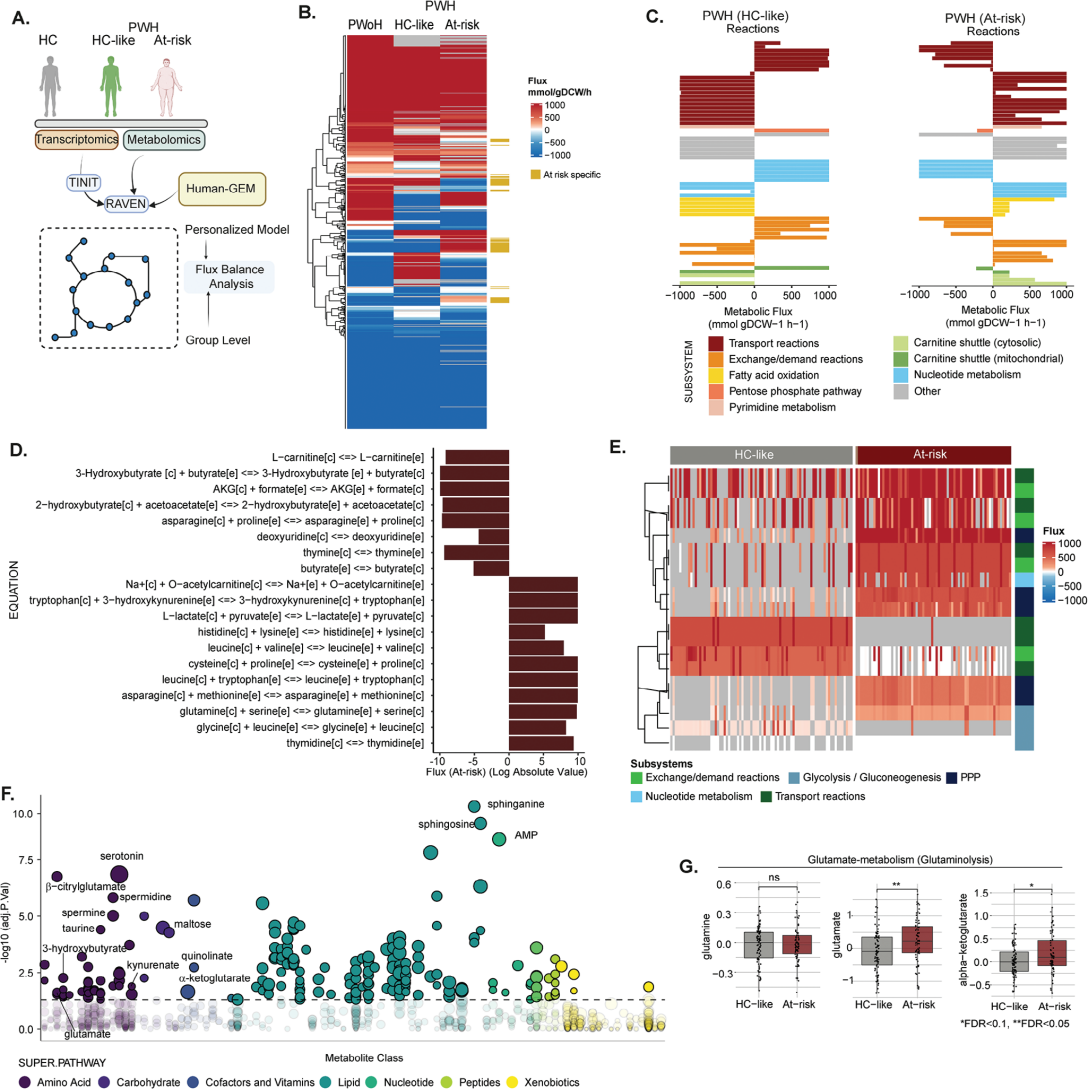
Modelling metabolism at the system level. A) Workflow describing the generation of groups and individual Genome‐scale metabolic models (GSMM) and further applications. B) Heatmap representing fluxes identified by flux balance analysis in grouped GSMM (HC, HC‐like, and at‐risk). Fluxes specific to at‐risk are labeled yellow. C) Barplot showing at‐risk‐specific fluxes separated based on subsystems in HC‐like (left) and at‐risk (right) GSMM. Fluxes with different directions have been included. Flux with values <250 were excluded. D) Barplot of at‐risk specific transport reactions based on flux values in at‐risk GSMM. Equations are indicated on the right. E) Heatmap representing fluxes values for each patient differing between HC‐like at at‐risk at the individual level based on Fisher test. The patient cluster is indicated above. Metabolon super pathways are indicated on the right. F) Dotplot of metabolites differing HC‐like and At‐risk. Size is inversely proportional to FDR. Dots are ordered based on Metabolon super pathways and sub‐pathways. Color is dependent on super pathways. The horizontal line represents –log10(0.05). G) Boxplot showing the levels of metabolites in glutamate‐metabolism.

The secretory microenvironment drives the major metabolic complication in the at‐risk PWH. Therefore, to identify the metabolic perturbations, we developed context‐specific genome‐scale metabolic (GSMM) models by integrating the transcriptomics and metabolomics from people without HIV infection (PWoH), HC‐like, and at‐risk PWH (**Figure** **4**A).^[^ ^9^ ^]^ We identified 64 unique metabolic fluxes in the at‐risk group (Figure 4B) that were either restricted to the at‐risk PWH only or had different directional flux than the HC‐like phenotype and PWoH (Table S8, Supporting Information). These risk‐specific metabolic reactions included transport reactions (*n* = 22), pentose phosphate pathway (*n* = 1), and fatty acid oxidation (*n* = 5), which are the components of the central carbon metabolism (Figure 4C). Several transport reactions were part of amino acid metabolism, carbohydrates, and fatty acid derivatives (Figure 4D), further supporting our secretome (Figure 2B). The personalized GSMM recapitulated the group‐level GSMM with critical fluxes related to central carbon metabolic pathways (FDR<0.1, Figure 4E) supported extracellular plasma metabolomics (FDR<0.1, Figure 4F and Table S9, Supporting Information). We have observed a significant increase in the host tryptophan pathway metabolites (serotonin, kynurenate, quinolinate), immune regulators [spermine (SPR) and spermidine (SPD)], and sphingolipids (sphinganine and sphingosine) in the at‐risk PWH, which indicates a state of inflammation and metabolic dysregulation that can lead to impaired neuroimmunometabolism (Figure 4F; Figure S5A, Supporting Information). Apart from the increase in the neurotransmitter serotonin, both α‐ketoglutarate and glutamate but not glutamine were also increased in the at‐risk population, indicative of increased glutaminolysis, which plays a critical role in neuroimmunometabolism and neurotransmission (Figure 4G).^[^ ^10^ ^]^ The RF‐based classification also identified the SPD, SPR, taurine, kynurenate, and serotonin as the top 30 metabolites (Figure S5B, Supporting Information). The microbial tryptophan pathway metabolites indole acetic acid also increased but were statistically not significant (LFC: 0.25, Adj *p* = 0.17). We also looked at our earlier fecal microbiome data^[^ ^1^ ^]^ in a subset of samples to identify the microbiome diversity and abundance in the two groups. However, no differences were observed in the 16s microbiota alpha or beta diversity, and the microbiota abundance (Figure S6, Supporting Information) suggests that the alterations in tryptophan metabolism likely originate from host‐related factors in at‐risk PWH. We also observed an increase in taurine, hydroxybutyrates, and acetoacetate (Figure S5A, Supporting Information), indicating significant liver stress further supported by a significant increase in alanine aminotransferase (ALAT) in at‐risk (30.5 vs. 22.5 IU L^−1^, *p* = 0.00024) (Table 1). The significant elevation of the immunoregulatory polyamines that regulate the balance between pro‐inflammatory and anti‐inflammatory responses in the central nervous system (CNS), the Kynurenine pathway of tryptophan metabolism metabolites (kynurenate and quinolinate), excitatory (glutamate), and modulatory (serotonin) neurotransmitters, along with increased glutaminolysis, may contribute to the modulation of neuronal activity and synaptic plasticity, potentially influencing neuroimmunometabolic functions in the at‐risk PWH.

### 2.5 Plasma Micro‐Environment Impairs Neuronal Function in Human Cortical Forebrain Organoids

**Figure 5:**
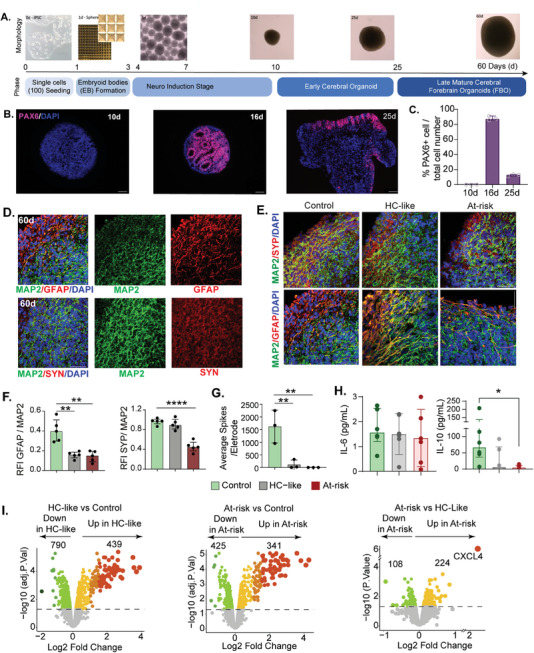
Neurological profiling and *ex vivo* assays in iPSCs differentiated functional cortical forebrain organoids A) Workflow for generating cortical forebrain organoids (FBOs) from human iPSC line with timeline. B) Representative confocal immunofluorescence images of human‐induced pluripotent stem cell‐derived cerebral organoids (COs) showing the expression of Paired Box 6 (PAX6) at 10, 16, and 25 days. C) A bar graph illustrating the percentage of PAX6‐positive cells in the CO at various time points during differentiation. D) Representative confocal immunofluorescence images of COs at 60 days demonstrating the presence of neurons (Microtubule Associated Protein 2, MAP2, green), astrocytes (Glial Fibrillary Acidic Protein, GFAP, red), and synapse (SYN, red) markers. E) Representative images of SYP (red) /MAP2(green) and GFAP(red)/MAP2(green) staining after the treatment of plasma from HC‐like and At‐risk. F) Quantitative analysis for SYP and GFAP expression normalized to MAP2 (*n* = 3). DAPI (blue) was used to visualize cell nuclei. G) 16‐electrode plates were used for Multi Electrode Array. MEA recordings, employing COs. Bar graph showing the average number of bursts per electrode across two‐month‐old FBOs maintained on MEAs from control and those treated by plasma from either HC‐like or At‐risk groups. One‐way ANOVA analyzed data sets with post hoc comparisons using Dunnett's multiple comparisons test compared to control samples. The stars above points represent Dunnett‐corrected post hoc tests. All data are presented as median (IQR) **p < 0.01; ***p < 0.001 ****p < 0.0001 versus control. H) Measurement of IL‐6 and IL‐10 in the supernatant exhibited by control (*n* = 6), HC‐like (*n* = 6), and At‐risk (*n* = 6) plasma‐treated FBOs. P‐value indicates Mann‐Whitney U test I) Volcano plots showing upregulated and down‐regulated proteins in iPSCs differentiated functional FBOs treated with HC‐like or at‐risk plasma. Scale bar = 50µm.

We hypothesized that the plasma micro‐environment with elevated polyamines, host‐tryptophan pathway metabolites, glutamate, and serotonin impacts neuronal activity and synaptic plasticity, leading to neuroimmunometabolic complications. As this is difficult to test in humans, we created 3D models of the human brain using pluripotent stem cells (iPSC) derived from cerebral forebrain organoids (FBOs), providing a valuable platform for studying the complex interactions between different cell types, including astrocytes and their role in neuroimmunometabolism. We used these FBOs because they constitute astrocytes upon maturation that play a significant role in neurotransmitter synthesis, metabolism, uptake recycling, neuronal metabolic support, neuroinflammatory response, and modulation of synaptic activity and plasticity. The process of the FBOs involved three stages: embryoid body formation, neuro induction, and maturation (**Figure** **5**A). To evaluate neuron differentiation and proliferation, we quantified the expression of the PAX6 marker in the organoids on days 10, 16, and 25 (Figure 5B). As expected, there were no PAX6‐positive cells at the early differentiation stage (10d). However, more PAX6‐positive cells were observed in the organoids at the intermediate stage (16d). At the mature stage (25d), PAX6‐positive cells were primarily located at the edges, indicating maturation of most organoids with continued growth at the periphery (Figure 5C). We performed immunostaining for mature neuronal marker Microtubule‐associated protein 2 (MAP2), astrocyte marker Glial fibrillary acidic protein (GFAP), and presynaptic marker synaptophysin (SYN) in day 60 organoids to assess the maturation and distribution of cells in the brain organoids (Figure 5D). We used these FBOs to determine the effects of the microenvironment on brain homeostasis and activity *ex vivo*. The level of the pre‐synaptic marker SYN was significantly low in at‐risk plasma‐treated FBOs but not in HC‐like, indicating a loss of synaptic density and function (Figure 5E). The level of astrocyte marker GFAP had significantly low expression (normalized to MAP2) in both HC‐like and at‐risk plasma‐treated organoids, further indicating defects in astrocyte‐mediated synapse transmission and neuroprotection. However, these astrocytes exhibited strong fibrous branches, suggesting reactive astrocytosis (Figure 5F). To identify the effect of the alterations of neuronal network properties arising from abnormal expression of synaptic proteins and activity, we used MEA recordings. Analysis of neuronal activity after plasma treatment at MEA showed a significant decrease in spike rates (Figure 5G) and bursting frequency (Figure S7A, Supporting Information) compared to the control, further supporting the reduced synaptic transmission deficits and altered network behavior. The IL‐10 secretion and relative mRNA level were significantly low in at‐risk plasma‐treated organoids (Figure 5H; Figure S7B, Supporting Information). No difference in IL‐6 secretion was observed between the HC‐like and at‐risk plasma‐treated monocytes. Finally, we performed quantitative proteomics to identify the FBOs' deep phenotyping following the microenvironment alterations. We identified significantly different levels (FDR<0.05) of several proteins while comparing to the control (Figure 5I and Table S10, Supporting Information) with upregulation of the process like apoptosis and regulation of autophagy in both HC‐like and at‐risk plasma‐treated FBOs (Figure S8 and Table S11, Supporting Information). While comparing the HC‐like plasma‐treated organoids with the at‐risk, only one protein (CXCL4) was significantly upregulated with FDR = 0.003 (Figure 5I) with several dysregulated proteins with significant nominal *p* < 0.05. Interestingly, the directionally based protein set enrichment analysis (PSEA) identifies the downregulation of OXPHOS (Table S11, Supporting Information), leading to an energy deficit, which can lead to synaptic degeneration, loss of synapses, and changes in synaptic structure.

### 2.6 SPD‐Enriched Microenvironment Reprograms Early Macrophage Polarization and Synaptic Function

**Figure 6:**
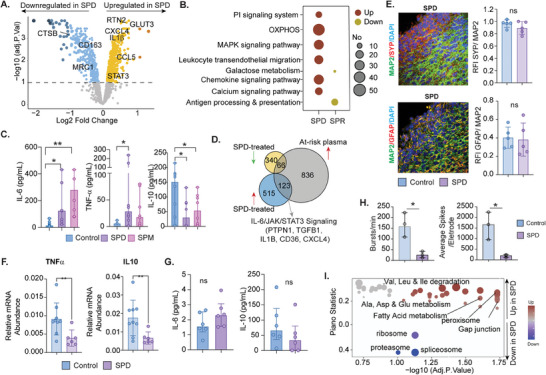
In vitro polyamines treatment in iPSCs differentiated functional cortical forebrain organoids. A) Volcano plots show the proteins differing between untreated monocytes and monocytes treated with spermidine B) Protein set enrichment analysis: Dotplots of pathways enriched in proteins differing untreated cells from cells treated with spermine and spermidine. The up‐regulation of pathways is indicated in red, and the down‐regulation in yellow. C) IL‐6, TNF‐a, and IL‐10 measurements in the supernatant. In the case of non‐detection, the lowest values for the respective kits were used. D) Venn diagram representing overlapping proteins between untreated cells and respective cells treated with spermidine and proteins differing cells treated with plasma from at‐risk patients. E) Representative images of SYP/MAP2 and GFAP/MAP2 staining after plasma treatment from HC‐like and at‐risk. In a quantitative analysis of SYP and GFAP expression normalized to MAP2, five random areas were selected to measure the fluorescence intensity. Scale bar =50µm.  F) The expression of TNFa and IL10 mRNA from cortical organoids from the control and SPD‐treated group (*n* = 3). G) Measurement of IL6 and IL10 in the supernatant. H) The average number of bursts per minute and electrode across two‐month‐old cortical organoids were maintained on MEAs treated with SPD or without any treatment. Data sets were analyzed by unpaired t‐tests. All data are presented as median (IQR) *p < 0.05; **p < 0.01; versus Control. I) Dot plot of KEGG pathways differing control organoids and organoids treated with spermidine. Val: Valine, Leu: Leucine, Ile: Isoleucine, Ala: Alanine, Asp: Aspartate, Glu: Glutamate.

As exogenous polyamines, SPD, and SPR were among the top high‐abundance metabolites in at‐risk PWH that have an immunomodulatory role, we hypothesized that a prolonged polyamines‐driven microenvironment impaired the macrophage functions. We treated the monocytes with polyamines SPD and SPR and activated them with lipopolysaccharides (LPS). Compared to the untreated cells, in SPD‐treated cells (**Figure** **6**A and Table S12, Supporting Information), several proteins involved in the metabolic processes [reticulon 2 (RTN2) facilitates glucose uptake, GLUT‐3 mediates glucose uptake, etc.], chemokine signaling pathway, [CCL5] and pro‐inflammatory process, [CXCL4, STAT3, IL1β] were upregulated. In contrast, proteins like Mannose Receptor C‐Type 1 (MRC1/CD206), CD163, and Cathepsin B (CSTB) were downregulated. As the level of CXCL4 in the plasma of the at‐risk PWH was higher (Figure 2A), we, therefore, posit an induction of M4 macrophage phenotype in the at‐risk PWH where CXCL4 modulates macrophage function by suppressing CD163.^[^ ^11^ ^]^ Interestingly, metabolic processes like OXPHOS were also upregulated, mainly characteristic of the anti‐inflammatory phenotype (Figure 6B). Though statistical significance was not reached due to high heterogeneity, a similar pattern was observed in the SPR‐treated cells (Figure S9, Supporting Information). In the supernatant, the levels of IL‐6 and TNF‐α were significantly higher in polyamine‐treated LPS‐activated monocytes (48 h post‐treatment) (Figure 6C). At the same time, IL‐10 was lower, further supporting the M4‐phenotype.^[^ ^12^ ^]^ While comparing the upregulated proteins in the SPD‐treated monocytes with at‐risk PWH's plasma profile, 123 proteins were overlapping, which was part of critical pathways for pro‐inflammatory conditions, including the activation of the IL‐6/JAK/STAT3 signaling cascade (Figure 6D). We treated the differentiated functional cortical neuronal organoids with SPD for 72 hrs to identify defects in synaptic transmission. There were no differences in the expression level of presynaptic protein SYN or astrocytes GFAP (Figure 6E) compared to the control (shown in Figure 5E). However, it changed the inflammatory balance as both TNFα and IL‐10 were significantly low (Figure 6F). It was also associated with changes in astrocytic morphology with astrocytic displaying activated phenotypes. Interestingly, the secretion of IL‐6 was slightly higher while IL‐10 was lower, although statistically, it was not significant in SPD‐treated organoids (Figure 6G). Analyzing neuronal activity following the SPD treatment by MEA showed decreased spike rates and bursting frequency (Figure 6H) compared to the control, indicating synaptic functional deficits in vitro. We also performed quantitative proteomics of the organoids. Though the differential protein expression did not find any significant proteins after corrections, the nominal p‐value identified the upregulation of 372 and the downregulation of 151 proteins (*p* < 0.05) (Table S13, Supporting Information). Though statistically not significant, both STAT3 and CXCL4 were higher in SPD‐treated cells. The directionality‐based protein set enrichment analysis identified upregulation of fatty acid, alanine, aspartate, and glutamate metabolism (FDR<0.2) (Figure 6I and Table S14, Supporting Information) that may influence neuronal energy metabolism.^[^ ^13^ ^]^ Combining all the data SPD and SPD‐driven microenvironment alters the macrophage polarization and synaptic transmission.

## 3 Discussion

In the present study, we have identified the significance of the plasma microenvironment in developing complex immune‐metabolic conditions in PWH undergoing prolonged antiretroviral treatment. Our findings revealed that myeloid cell lineage‐driven secretory plasma microenvironment impaired monocyte function in those immunometabolically dysregulated PWH. We identified five biomarkers of immunometabolic dysregulation linked to metabolic processes. Interestingly, within this microenvironment, distinct reprogramming occurs in various cell types differently, with significant functional alterations observed primarily in monocytes rather than T‐cells (CD4 and CD8). Moreover, the plasma microenvironment in the at‐risk group impaired the differentiated functional FBOs *ex vivo*, which can lead to synaptic dysfunction and alterations in neuronal signaling and adult neurogenesis.^[^ ^14^ ^]^

The microenvironment exquisitely regulates monocyte/macrophage function. The plasma secretome and pathway analysis indicated stress‐mediated metabolic reprogramming in the at‐risk PWH. It regulates cell survival and proliferation due to the myeloid cell lineage secretory SAP. It created the exhausted phenotype, leading to impaired macrophage function. Among the top five biomarkers, GGCT is involved in glutathione metabolism, which protects cells from oxidative stress and other cellular damage.^[^ ^15^ ^]^ TBCB, tubulin‐binding cofactor (TBC), plays a vital role in the assembly of the microtubules essential in regulating several metabolic processes and may affect cell proliferation and migration.^[^ ^16^ ^]^ LATS1 also attenuates mTORC1 kinase activation,^[^ ^17^ ^]^ which regulates critical metabolic processes and controls cell proliferation. Moreover, in our RF analysis, several essential proteins were part of the metabolic processes like glycolysis/gluconeogenesis (Enolase 1; ENO1), amino acid metabolism (Kynurenine—oxoglutarate transaminase 1; KYAT1), glucose and energy metabolism (Fatty Acid Binding Protein 5, FABP5) and were linked to the mammalian target of rapamycin (mTOR) pathway (Calcyclin Binding Protein; CACYBP, General vesicular transport factor p115; USO1, Eukaryotic translation initiation factor 4 gamma 1; EIF4G1, and ENO1) and cellular responses to stress, e.g., DnaJ Heat Shock Protein Family (DNAJB6 and DNAJA2), FKBP Prolyl Isomerase 5 (FKBP5), Heat Shock Protein Family A (Hsp70) Member 1A (HSPA1A). In the functional assay after treating the monocytes of healthy donors with the at‐risk plasma, the downregulation of TOMM22 indicated impaired mitochondrial respiration and energy production, including impaired protein import and oxidative phosphorylation, leading to dysfunctional host metabolism.^[^ ^18^ ^]^ The functional stimulation assays of the monocytes and T‐cells further supported it. Exposure to the at‐risk plasma of the healthy showed a trend in higher expression for the CCR2 and CX3CR1, indicating activation of the monocytes due to the at‐risk plasma treatment. The higher expression of CD86 and the lower expression of CD38 and PDL1 in the at‐risk plasma‐treated cells also indicate impaired interaction with the T‐cells and potential exhaustion of the monocytes that impact the polarization and trafficking of the monocytes.^[^ ^19^ ^]^ Further downregulation of the NRP2 in at‐risk treated monocytes can lead to impaired migration and homing of monocytes to sites of inflammation, as NRP2 acts as a co‐receptor for several chemokines.^[^ ^20^ ^]^ Interestingly, the plasma microenvironment from the at‐risk population does not impair the T‐cell polyfunctionality. The imbalance between T‐cell and monocyte function could lead to dysregulated inflammatory responses, potentially increasing the risk of chronic inflammatory disorders within the at‐risk population.

The host tryptophan metabolism was severely impacted in the at‐risk PWH. The FBA analysis predicted an extracellular flux of tryptophan from the cytoplasm to extracellular space through the kynurenine pathway. An increased flux was also detected for amino acids (AA), indicating a dysregulated AA pathway in the at‐risk PWH, leading to imbalances in the levels of AA and dysregulated energy metabolism. The plasma metabolomics further indicated the prominent dysregulation of the tryptophan metabolism, as serotonin, kynurenate, and quinolinate levels were high in the at‐risk population because of the inflammatory environment due to the tryptophan breakdown. An increase in kynurenate and polyamines (e.g., SPR and SPD) might exert an immunoregulatory effect on the monocyte functions as SPD has been shown to modulate indoleamine 2,3‐dioxygenase 1 (IDO1) enzyme converts tryptophan to kynurenine pathway metabolites.^[^ ^21^ ^]^ Importantly, the tryptophan metabolism through the kynurenine pathway in the liver can affect liver health, as kynurenine and its downstream metabolites can affect immune function and inflammation, which are involved in liver disease through the gut‐liver axis. Higher ALAT in the at‐risk PWH further supports it. The increased levels of hydroxybutyrate and acetoacetate in the blood indicate a state of ketosis that occurs when the body switches from using glucose as its primary energy source to ketones, produced from fatty acids.^[^ ^22^ ^]^ The tryptophan metabolism through the serotonin pathway can also affect liver function.^[^ ^23^ ^]^

Increased serotonin is a neurotransmitter that plays a role in mood regulation and gastrointestinal function and can affect lipid metabolism and inflammation. It could be regulated by polyamine metabolism as it plays a crucial role in liver function. The gut‐liver‐brain axis, a multidirectional communication network linking the enteric, hepatic, and central nervous systems, may shape the immune‐metabolic status of the at‐risk PWH through systemic communication. Moreover, the peripheral plasma microenvironment can contain inflammatory mediators that can cross the blood‐brain barrier and directly impact the function of central nervous system (CNS) cells.^[^ ^24^ ^]^ This can lead to neuroinflammation and activation of immune cells within the CNS, contributing to neuronal dysfunction and damage. In individuals with Alzheimer's disease (AD), peripheral inflammation escalates amyloid beta levels in the brain,^[^ ^25^ ^]^ potentially due to increased blood‐to‐brain influx and decreased brain‐to‐blood efflux across the blood‐brain barrier.^[^ ^26^ ^]^ Subsequently, it can foster neuroinflammation and disease advancement.^[^ ^27^ ^]^ While treating the brain organoid with the at‐risk and HC‐like plasma, the only protein significantly upregulated with FDR<0.05 in at‐risk plasma‐treated organoid was CXCL4 when compared to the HC‐like plasma. The CXCL4 induces the M4 macrophages characterized by lower expression of the CD163 and higher expression of CD86 but can secrete higher levels of IL‐6 and TNFα and lower levels of IL‐10.^[^ ^12^ ^]^ In our plasma secretome data, we observed a higher level of CXCL4. While the PBMCs were treated with pooled plasma from at‐risk PWH, we observed a higher expression of CD86 in the macrophages.

Moreover, the level of presynaptic marker Synaptophysin (SYN) was decreased in the at‐risk plasma‐treated organoids, indicating that a defect in synapse development may lead to synaptic dysfunction and alterations in neuronal signaling and adult neurogenesis.^[^ ^14^ ^]^ This is further supported by alterations of the several synaptic proteins identified in the proteomics data. Interestingly, the directionally based PSEA identified the downregulation of OXPHOS in at‐risk plasma‐treated organoids compared to HC‐like treated, which can lead to energy depletion, cell death, neurotoxic effects, and reduced neuronal activity, potentially contributing to age‐related decline.^[^ ^28^ ^]^

The study has limitations that merit comments. First, though this is the most extensive multi‐omics study involving 158 PWH, it may not fully represent the diversity within the population of PWH. A larger and more diverse sample could provide a more comprehensive understanding of immunometabolic impairments. Second, the study population was over‐represented by Caucasian males. The study may not have fully accounted for ethnic and genetic variations in the study population, which can play a significant role in immunometabolic responses. Third, while five plasma biomarkers were discovered, the study design does not fulfill the classical biomarkers discovery study. Additional studies are needed to validate these biomarkers in larger and more diverse populations to confirm their utility as predictors of immunometabolic complications in PWH. Fourth, the 3D organoids recapitulate complex 3D brain structures, which allows for investigating the development and function of specific forebrain regions implicated in neuropsychiatric disorders. However, it is essential to acknowledge that no model system is perfect, and organoids are no exception. Existing brain organoids, for example, have limitations such as the absence of vascular and immune cells and other non‐neural cells. While neuronal populations in organoids exhibit diverse layer molecular signatures of the cortical plate, they cannot reproduce the six‐layered spatial organization in human brains. Finally, the study focuses on identifying risk factors and mechanisms but may not directly address clinical outcomes or interventions. Future research could explore the translation of these findings into clinical practice.

## 4 Conclusions

In summary, we discovered the importance of the endogenous plasma inflammatory microenvironment responsible for chronic inflammation in the prolonged treated immunometabolically complicated at‐risk PWH. The immunometabolically compromised at‐risk PWH has a higher risk of cardiovascular and neuropsychiatric disorders driven by the gut‐liver and gut‐brain axis. Earlier identification of the at‐risk PWH based on the biomarker identified in our study potentiates early intervention to improve the inflammatory condition. Our study further emphasized the significant impact of dysregulated tryptophan metabolism through the kynurenine pathway in the at‐risk population. It could lead to imbalances in amino acid levels and energy metabolism, elevating serotonin, kynurenate, and quinolinate levels due to the inflammatory environment caused by tryptophan breakdown. Furthermore, this chronic inflammatory plasma microenvironment in the at‐risk PWH contributed to synaptic dysregulation *ex vivo*. This disruption subsequently increases the likelihood of neurological and psychiatric symptoms in these individuals through perturbations in neuroimmunometabolism.

## 5 Experimental Section

### Study Population

The study includes 158 PLWH from the Copenhagen comorbidity in HIV infection (COCOMO) cohort^[^ ^29^ ^]^ and 18 HIV‐negative controls (HC). It all originated from the Copenhagen area. Clinical and demographic data were retrieved from the COCOMO database. Ethical approval was obtained by the Regional Ethics Committee of Copenhagen (COCOMO: H‐15017350) and Etikprövningsmyndigheten, Sweden (Dnr: 2022‐01353‐01). Informed consent was obtained from all participants and delinked before analysis.

### RNA Sequencing (RNAseq)

Total RNA was extracted from the peripheral blood mononuclear cells (PBMC) using the Quick‐RNA™ Miniprep Plus Kit (Zymo Research). The library was prepared using the Illumina TruSeq Stranded mRNA (Illumina). Samples were sequenced on NovaSeq6000 (NovaSeq Control Software 1.7.5/RTA v3.4.4) with a 151nt(Read1)‐10nt(Index1)‐10nt(Index2)‐151nt(Read2) setup using “NovaSeqXp” Workflow in “S4” mode flowcell at National Genomics Infrastructure Sweden (NGI).

### Plasma Secretome Analysis

It was used Olink® Explore, a multiplex immunoassay protein biomarker platform that detects ≈3000 proteins in plasma. The platform uses Proximity Extension Assay (PEA) technology run on Illumina NovaSeq 6000 system (Illumina, US). The list of proteins was given in Table S12 (Supporting Information). Detailed methodology was presented in supplementary materials and methods.

### Plasma Metabolomics

Plasma untargeted metabolomics was performed at Metabolon, Inc. (North Carolina, USA), as previously described.^[^ ^5^ ^]^ Briefly, recovery standards were added to 100 µL of plasma prior to sample preparation using the automated MicroLab STAR® system (Hamilton Company, USA). The resulting extract was divided into four fractions: two for analysis using separate reverse‐phase (RP) UPLC‐MS/MS methods with positive ion mode electrospray ionization (ESI), one for RP/UPLC‐MS/MS analysis with negative ion mode ESI, and one for HILIC/UPLC‐MS/MS analysis with negative ion mode ESI. The mass spectrometry (MS) analysis alternated between MS and data‐dependent MSn scans, with the m/z range slightly varying between methods but generally covering 70–1000 m z^−1^. Data was normalized to sample volume, log‐normalized, and minimum‐imputed as given by Metabolons^TM^’s proprietary pipeline. The metabolomics method was ISO 9001:2015 certified, and the lab was accredited by the College of American Pathologists (CAP), USA. The detailed method was published elsewhere.^[^ ^30^ ^]^

### Supernatant Protein Analysis

The individual proteins were measured from the supernatant of the *ex vivo* assays either by ProQuantum Immunoassay Kit (IL6 and IL10) (Invitrogen, USA) where the sample volume was low or by Human Quantikine® ELISAs (IL6, IL10, TNF‐α) (R&D Systems, USA) where the volume was high. The kit catalog numbers were provided in Table S13 (Supporting Information).

### Similarity Network Fusion (SNF)

Genes, proteins, and metabolites with variance<0.2 were removed from the data. Patient clustering was performed using SNFtool as described^[^ ^31^ ^]^ and the three data layers. Data were standard normalized for each layer, pairwise sample distances were calculated, and a similarity network was built (number of neighbors, K = 30, hyperparameter, alpha = 0.7). Networks were fused into similarity network fusion (K = 30, number of iterations, T = 20), and spectral clustering was applied to identify the optimal number of clusters (Clusters, C = 2). Parameters were selected based on maximum eigengap and minimum rotation cost. Concordance between fused networks and individual networks was reported in normalized mutual information (NMI).

### Clusters Validation with netDx

Multi‐omics and clinical data were used as input for netDx model.^[^ ^32^ ^]^ The type of comparison was selected as pearsonCorr for omics and normDiff for clinical data. The proportion of samples to train the model was set to 0.8, the number of train/tests splits to 20, and the threshold for feature selection was seven or more out of a max score of 10 in 70% of splits. Model performance was verified using receiver operating characteristic (ROC) and precision‐recall (PR) curves produced by the getResults function and a merged confusion matrix made by the confusionMatrix function.

### Gene Set Enrichment Analysis

Pathway analysis was performed using Cystoscape module The Biological Networks Gene Ontology tool (BiNGO) and Gene Ontology terms, R package PIANO using KEGG metabolism terms, and MSigDB terms for proteins and genes. For metabolites, enriched pathways were described using python module gseapy and metabolon terms. Terms with FDR<0.2 were considered significant.

### Feature Selection using Random Forest

Using proteomics and metabolomics data, Radom Forest was performed to predict biomarkers that differentiate clusters. First, consensus feature selection was performed using the R package Boruta by randomly sampling 1000 proteins for 1000 iterations. Proteins were identified in 70% of iterations, where they were selected and kept as the most relevant features. To validate these features, the final random forest model was built using node size as default, 700 trees, three repetitions of 10‐fold cross‐validation, and a test for several predictors randomly sampled at each split (mtry) between 1 and 15. The model was evaluated using a confusion matrix and accuracy measure based on the put‐of‐bag (OOB) estimate of the error rate.

### Structural Causal Modelling

Bayesian belief networks (BBNs) were computed based on the 187 top proteins differing conditions using the bnlearn package with a score‐based hill‐climbing algorithm that searches all possible directed acyclic graphs (DAGs).^[^ ^33^ ^]^ The importance of each DAG was measured with the maximization of the Bayesian information criterion (BIC) score. A final consensus network was built based on 150 random networks and followed by the removal of undirected edges. Then, edges were permutated to refine the network (iterations = 1000). Each gene was removed, and the change of BIC between the new network and the consensus network was used to identify driver genes, which induced the highest decrease in BIC after being removed. Then to validate driver genes, BIC change between the network without the driver genes and five random genes was performed for 1000 iterations.

### Consensus Association Analysis

400 random features from each data set were selected and combined per iteration (iterations, I = 1000). Pairwise Spearman correlations were performed, and significant correlations (FDR<10^−6^) were kept. Significant associations between two features found in 90% of the iterations, including this association, were kept building a co‐expression network. The correlation between consensus associations was re‐calculated, and associations with FDR<0.00005 were used to build negative and positive networks using python igraph.^[^ ^34^ ^]^ Networks were compared to random networks of the same size. Highly interconnected features were clustered using leiden algorithm from the leidendag python module into communities.^[^ ^35^ ^]^ The mean degree was calculated per community. Only communities with more than 30 features were used.

### Senescent Markers

Genes from CellAge senescence database (*n* = 1259, https://genomics.senescence.info/cells/), Csgene senescence database (*n* = 504, http://csgene.bioinfo‐minzhao.org/) and Reactome SASP (*n* = 81, https://reactome.org/content/detail/R‐HSA‐2559582) were retrieved.

### Cell Profiling and Single‐Cell Data Analysis

CIBERSORTx was used to identify immune cell proportions from bulk RNA‐seq data for each patient using a gene signature matrix (LM22) which contains 547 genes differing from cell types.^[^ ^36^ ^]^ The single‐cell RNA sequencing (scRNAseq) data were retrieved from 10xGenomics as a count file from one patient (https://www.10xgenomics.com/resources/datasets/20‐k‐human‐pbm‐cs‐3‐ht‐v‐3‐1‐chromium‐x‐3‐1‐high‐6‐1‐0) Seurat was used for downstream analysis (https://satijalab.org/seurat/). The Seurat object was created with features detected in a minimum of 3 cells, and cells with 200 features were counted as minimum and 2500 as maximum. Cells were removed if they contained >7% of the mitochondrial count. Normalization using the global‐scaling normalization method and identification of variable features was performed using default parameters. Before visualization, linear transformation was applied to data. To reduce dimensionality, principal component analysis (PCA) was performed. Principal component cut‐offs were determined based on the JackStraw plot and used for the rest of the analysis. Data visualization was performed using t‐distributed stochastic neighbor embedding (tSNE). The Graph‐based clustering approach was performed using the Louvain algorithm and a resolution parameter of 0,5. Cell type identification was performed using canonical markers extracted from Seurat. Differential gene expression analysis was performed between clusters and cell types identified using the FindAllMarkers function with the Wilcoxon Rank‐Sum Test. The p‐values were adjusted for multiple hypotheses using Benjamini and Hochberg correction. Significant genes were kept if FDR<0.05.

### Genome‐Scale Metabolic Models

The genome‐scale metabolic models (GSMM) model analysis was performed using RAVEN v2.4 (https://github.com/SysBioChalmers/RAVEN). RAVEN needs MATLAB installation, libSBML libraries, and a Gurobi solver. Models were reconstructed from the template Human‐GEM (https://github.com/SysBioChalmers/Human‐GEM) by integrating RNA‐seq data in TPM format using the function getINITModel2 and modifying boundaries of transport reactions of metabolites using metabolomics data. Models were kept if able to perform the 21 essential tasks using the checkTasks function. Flux balance analysis for each model was performed, with the object being biomass production. Fluxes were filtered out if inferior to 10^−7^ mmol gDCW^−1^h^−1^, and for visualization purposes, fluxes with a value inferior to 250 were removed from the figures. Transport flux was also log2 transformed for easier visualization.

### T‐Cell Functionality Assay

The donor PBMCs (*n* = 6) were incubated with 15% pooled plasma from HC‐like (*n* = 10) and at‐risk (*n* = 10) separately and stimulated with Cytomegalo‐, Epstein‐Barr, and Flu‐virus (CEF) peptide pool (2ug/mL), or left unstimulated, in RPMI (10 mM HEPES, 2 mM L‐glutamine, and 0.1% PenStrep) supplemented with DNase I recombinant, RNAse‐Free (Roche, USA) for 48 h. The inhibitors Brefeldin A (GolgiPlug, BD bioscience, USA) and Monensin (GolgiStop, BD bioscience, USA), were added 12 h before collecting the samples. Samples were collected and washed in FACS buffer (PBS+2%FBS+2mM EDTA) before staining of anti‐CCR7 for 10 min at 37 °C and 5% CO_2_ following staining of additional surface receptors and aqua viability stain (Thermofisher) for 30 min at room temperature (RT). Fixation and permeabilization were performed using FoxP3/transcription factor staining buffer set (Thermofisher, USA) for 30 min at room temperature (RT). Fixation and permeabilization were performed using FoxP3/transcription factor staining buffer set (Thermofisher) following intracellular staining for 30 min at RT. After additional washing, the samples were immediately run on FACS symphony (BD Bioscience, USA). Analysis was performed in FlowJo v.10.8.1 (FlowJo, LLC). The gating strategy was detailed in Figure S4A (Supporting Information). The stimulation index represents the fold change relative to unstimulated PBMCs incubated with healthy control plasma (negative control). Phenotypic analysis was performed using Boolean combination gates of the frequencies for functional markers (Granzyme B and Perforin), activation markers (CD27, CD38, CD69, CD226, HLA‐DR, and Ki‐67), and exhaustion markers (CTLA‐4, Eomes, KLRG1, PD1, T‐Bet, TIGIT, Lag3, and TIM‐3) separately in FlowJo and visualized using Simplified Presentation of Incredibly complex evaluation (SPICE) version 6.1 (https://niaid.github.io/spice/). The reagent catalog numbers were provided in Table S13 (Supporting Information).

### Monocyte Functionality Assay

The donor PBMCs (*n* = 6) were incubated with 50% pooled plasma from HC‐like (*n* = 10) and at‐risk (*n* = 10) separately and stimulated with LPS (1 pg mL^−1^) or unstimulated in RPMI (10 mM HEPES, 2 mM L‐glutamine, and 0.1% PenStrep) for 48 h. Samples were collected and washed in FACS buffer (PBS+2%FBS+2mM EDTA) and staining of surface receptors and near‐IR viability stain (Invitrogen) for 30 min at 4 °C. After fixation (2% PFA), samples were run on FACS symphony (BD Bioscience). Phenotypic analysis was performed using Boolean combination gates of the frequencies for expression of chemokine receptors (CCR2, CCR5, and CX3CR1), activation markers (CD38 and CD86), and expression of PDL1+ on monocytes. The gating strategy was detailed in Figure S4C (Supporting Information).

### Spermidine and Spermin Stimulation

Monocytes were isolated from donor PBMCs (*n* = 4) using EasySep™ Monocyte Isolation Kit (Stemcell, #19 359) and seeded with 1×10^6^ cells mL^−1^ density. Monocytes were incubated in RPMI (10% FBS, 5% human AB serum, 10 mM HEPES, 2 mM L‐glutamine, and 0.1% PenStrep). 3 h before the cells were treated with either SPD (35 µM, Sigma–Aldrich, USA) or SPR (10 µM, Sigma–Aldrich, USA) or untreated. The dose was selected based on the cell viability where at least 70% of the cells were viable. After an hour, macrophages were stimulated with LPS(1 pg mL^−1^) and incubated for 48 h. For proteomics, samples were collected and washed in FACS buffer (PBS+2%FBS+2mM EDTA). Cell numbers were determined, and cells were washed with PBS. The supernatant was removed completely, and samples were stored at −80 °C until analysis.

### Organoid Differentiation

Human iPSCs Sli021 were differentiated into cortical organoids. The iPSCs cells were collected and filtered by 37 µm Reversible Strainer (STEMCELL) to get single‐cell suspension. The suspension was transferred to AggreWellTM 400 plates (STEMCELL) to generate embryoid bodies (EBs) with EB Formation Medium (STEMCELL). Next day, the harvesting Human EBs were transferred to 6‐well low‐attachment plate and cultivated in Neuron Induction Media (Neurobasal media, 1% 100x GlutaMAX, 1% Penicillin/streptomycin, 1% 100x N2 supplement, 2% 50x B27 supplement, 1.25 uM dorsomorphin, 10 ng ml^−1^ human recombinant LIF, 3 uM CHIR99021 and 3 uM SB431542). After 10 days, the organoids were maintained in the organoids medium (50% DMEM/F‐12, 50% Neurobasal media, 1% Penicillin/streptomycin, 0.5% 100x N2 supplement, 1% 50x B27 supplement, 1 ug ml^−1^ Humulin, 200 nM 2‐Mercaptoethanol, 1% 100x GlutaMAX, 0.5% 100x Minimum Essential Media (MEM), 20 ng ml^−1^ BDNF, 20 ng ml^−1^ GDNF and 20 ng ml^−1^ FGF2) for 7 weeks.

### Multiple Electrode Arrays (MEAs) Assays

MEA plates were pre‐treated with 10 ul drop of 0.1% PEI solution and incubator at 37 °C, 5% CO_2_ for at least 60 min. 7‐week‐old organoids were plated in the MEA plate with 10 ul organoids medium containing 10 µg ml^−1^ laminin and make sure the organoids covered all the electrode arrays. After 1 h at 37 °C, 5% CO_2_ incubation, 500 ul medium was added into each MEA well and the medium was half‐changed every day for 7 days. SPD and Plasma from HC‐like and At‐risk were added into the medium and treated for 3 days. The electrophysiological activity was recorded after 3‐day's treatment using hardware (Maestro Pro complete with Maestro 768‐channel amplifier) and software (AxIS 1.5.2) from Axion Biosystems (Axion Biosystems Inc., Atlanta, GA). All other parameters were defaulted and the temperature and CO_2_ concentration were 37 degrees and 5% respectively. Raw data were analyzed by MATLAB with custom scripts. The signal activities from all electrodes were involved in this analysis. Three duplicate MEAs/organoids for each group were performed for the analysis.

### Gene Expression Analysis

RNA was isolated from cortical organoids using a GeneElute™ mammalian total RNA miniprep kit (Sigma). After normalizing the RNA concentration to ≈50 ng ul^−1^ using nuclease‐free water (Promega), a high‐capacity cDNA reverse transcription kit (Applied Biosystems) was used for creating cDNA. For quantitative real‐time PCR (qPCR), 70 ng of cDNA was used per reaction, and primer sequences are in the Oligonucleotides parts of key resources. StepOnePlus™ real‐time PCR system (Applied Biosystems) with qPCRBIO SyGreen Blue Mix Hi‐ROX (PCR Biosystems) was used to produce PCR products detecting by incorporation of SYBR‐green and authenticated by the melt‐curves. All the results were normalized to GAPDH presenting in terms of the 2^−ΔΔCT^ method or relative mRNA abundance.

### Organoids Cryosection and Immunohistochemistry (ICC)

The organoids were fixed in ice‐cold 4% PFA, pH = 7.4 for 15–20 h and washed 3 times in PBS. 30% sucrose solution was used to remove the water in organoids until the COs no longer float. Embedding solution (50% O.C.T Compound + 50% of 30% sucrose solution) was involved to embed organoids on dry ice. After organoids were frozen at −80 °C for 15 h, they were cryosectioned into 15 µm sections on a Small Lecia cryosection. The sections were then blocked and permeabilized using 5% goat serum in PBS with 0.1% triton‐X‐100 (PBST). Primary antibodies (SYP, Sino bioscience; GFAP, Dako; and MAP2, NOVUS) were used in the blocking solution and incubated at 4 °C overnight. Secondary antibodies were diluted in PBST and applied for 2 h at room temperature. Cells were counterstained with DAPI and mounted using ProLong™ Glass Antifade Mountant (Thermo Fisher). Relative fluorescence intensity was quantified using Image J using five random fields per organoid. The images were taken by a Leica DMI600B inverted time‐lapse microscope or ZEISS Axio Scan.Z1 with a 20x objective.

### Single‐Cell Type Quantitative Proteomics

Single‐cell type quantitative LC‐MS/MS‐based proteomics was performed as described previously.^[^ ^4a^ ^]^ Raw data were filtered for missing values and quantile normalized. Differential abundance analysis was performed using LIMMA. Models were corrected for replicates and the number of cells. *P* values were adjusted using Benjamini‐Hochberg (BH) correction.

### Visualization

R package ggplot2^[^ ^37^ ^]^ was used to design boxplots, bubble plots, volcano plots, importance plots, PCA plots, scatter plots, bar plots, and dot plots, while ggalluvial was used to make the Sankey plot. Cytoscape v.3.6.1^[^ ^38^ ^]^ was used to represent large networks. Heatmaps were made using the R package ComplexHeatmap. ROC curves were obtained with the getResults function from the netDx package. The t‐SNE plots were produced by the Seurat function Dimplot. Biorender was used to make Workflow (https://biorender.com/).

### Statistical Analysis

Continuous clinical variables were compared using a t‐test if normally distributed or a Mann‐Whitney U test if not normally distributed. Discrete variable differences were tested using the Chi‐Square Test if the expected values of the contingency table were five or more; otherwise, Fisher's Exact Test was applied. Spearman correlations between features were performed using the function rrcor from the R package Hmisc. Proteomics and metabolomics were analyzed with R package limma for differential abundance analysis. The difference in gene expression was investigated using R package DESeq2.^[^ ^39^ ^]^ Three models were designed for each data set, including HC and HIV (model 1), between HIV clusters (model 2) and comparing clusters and corrected for confounding variables (model 3). All test *P* values were adjusted using Benjamini‐Hochberg (BH). The default p‐value cut‐off was set to 0.1. Other p‐values cut‐offs are adapted for a specific analysis and indicated. For wet lab experiments, statistical analysis was performed using Graphpad prism 9.0 (Graphpad Software Inc). Non‐parametric tests were used for analyzing the data where paired groups were compared using a two‐tailed Wilcoxon matched‐pairs signed rank test or Friedman test for multiple comparisons. The Permutation test was used to analyze cell populations visualized by SPICE version 6.1.

## Conflict of Interest

O'Mahony has served on speaker bureaus for Abbott, Nestlé, Nutricia, Reckitt and Yakult; received research funding from Chiesi and GSK; and acted as a consultant for PrecisionBiotics. MR is an Olink Proteomics, Boston, Massachusetts, United States employee. VP was an employee of Olink GmbH, Munich, DE, when the study was conducted and is presently Novo Nordisk, Denmark employee. RB was an employee at NBIS at the time of data analyses. RB is currently an employee of Chiesi Farmaceutici S.p.A. and does not hold Chiesi Farmaceutici S.p.A. stocks or equity shares. Chiesi Farmaceutici S.p.A. was not involved with the current study. Others none to declare.

## Author Contributions

F.M, and M.G contributed equally to this work. U.N. performed conceptualization. F.M., M.G., M.R., M.T., A.V., Y.A.S., R.B., and U.N. performed methodology. F.M., M.G., A.E., T.W., S.G., A.O., S.S.A., S.S., P.N., V.S., N.N., M.V., A.D.K., B.V., J.H., and J.R.H. performed investigation. F.M. T.W., A.O., A.E., and U.N., performed visualization. U.N., S.D.N., and Y.A.S., performed funding acquisition. U.N., S.D.N., and Y.A.S., performed project administration. U.N., S.D.N., A.V., R.B., M.R., Y.A.S., and A.C.K., performed supervision. T.B., M.T., V.P., M.R., R.B., L.O.M., R.S., N.K.B., M.V., J.P.M., S.W., and A.M. provided critical review of the manuscript. F.M., T.W., M.G., Y.A.S., and U.N., wrote the original draft. W.E., A.O., S.S.A., V.S., and A.D.K. wrote, reviewed and edited.

## Supporting information



Supporting Information

Supplemental Table 1

## Data Availability

The raw RNAseq data have been deposited in the NCBI/SRA with PRJNA983231. All the codes are available at GitHub: https://github.com/neogilab/Immunometabolism_HIV.The mass spectrometry proteomics data have been deposited to the ProteomeXchange Consortium (http://proteomecentral.proteomexchange.org) via the PRIDE partner repository with the dataset identifier PXD043296. The clinical, lifestyle, and demographic data is confined to the study group, but the request for data can be submitted to cocomo.rigshospitalet@regionh.dk.

## References

[1] F. Mikaeloff , M. Gelpi , R. Benfeitas , A. D. Knudsen , B. Vestad , J. Hogh , J. R. Hov , T. Benfield , D. Murray , C. G. Giske , A. Mardinoglu , M. Troseid , S. D. Nielsen , U. Neogi , eLife 2023, 12, 82785. 10.7554/eLife.82785 PMC10017104 36794912

[2] a) E. Brunet‐Ratnasingham , M. Dube , D. E. Kaufmann , Trends Mol. Med. 2019, 25, 1; 30473188 10.1016/j.molmed.2018.11.001

[3] S. S. Akusjarvi , U. Neogi , Curr. HIV/AIDS Rep. 2023, 20, 42. 36695947 10.1007/s11904-023-00646-0 PMC10102129

[4] a) S. S. Akusjarvi , A. T. Ambikan , S. Krishnan , S. Gupta , M. Sperk , A. Vegvari , F. Mikaeloff , K. Healy , J. Vesterbacka , P. Nowak , A. Sonnerborg , U. Neogi , iScience 2022, 25, 103607; 35005552 10.1016/j.isci.2021.103607 PMC8718889

[5] M. Gelpi , F. Mikaeloff , A. D. Knudsen , R. Benfeitas , S. Krishnan , S. Svenssson Akusjärvi , J. Høgh , D. D. Murray , H. Ullum , U. Neogi , S. D. Nielsen , Aging 2021, 13, 22732. 34635603 10.18632/aging.203622 PMC8544298

[6] B. Kelly , E. L. Pearce , Cell Metab. 2020, 32, 154. 32649859 10.1016/j.cmet.2020.06.010

[7] R. M. Kratofil , P. Kubes , J. F. Deniset , Arterioscler., Thromb., Vasc. Biol. 2017, 37, 35. 27765768 10.1161/ATVBAHA.116.308198

[8] S. Svensson Akusjarvi , S. Krishnan , A. T. Ambikan , F. Mikaeloff , S. Munusamy Ponnan , J. Vesterbacka , M. Lourda , P. Nowak , A. Sonnerborg , U. Neogi , AIDS 2023, 37, 1023. 36779490 10.1097/QAD.0000000000003512 PMC10155691

[9] A. T. Ambikan , H. Yang , S. Krishnan , S. Svensson Akusjärvi , S. Gupta , M. Lourda , M. Sperk , M. Arif , C. Zhang , H. Nordqvist , S. M. Ponnan , A. Sönnerborg , C. J. Treutiger , L. O'Mahony , A. Mardinoglu , R. Benfeitas , U. Neogi , Cell Syst. 2022, 13, 665. 35933992 10.1016/j.cels.2022.06.006 PMC9263811

[10] V. Gkini , T. Namba , Neurol. Psychiat. 2023, 29, 177. 10.1177/10738584211069060 PMC10018057 35057642

[11] G. Domschke , C. A. Gleissner , Cytokine 2019, 122, 154141. 28899579 10.1016/j.cyto.2017.08.021

[12] C. A. Gleissner , I. Shaked , K. M. Little , K. Ley , J. Immunol. 2010, 184, 4810. 20335529 10.4049/jimmunol.0901368 PMC3418140

[13] F. Tescarollo , L. Covolan , L. Pellerin , Front. Neurosci. 2014, 8, 246. 25161606 10.3389/fnins.2014.00246 PMC4130107

[14] W. Kelsch , S. Sim , C. Lois , Annu. Rev. Neurosci. 2010, 33, 131. 20572770 10.1146/annurev-neuro-060909-153252

[15] Z. He , X. Sun , S. Wang , D. Bai , X. Zhao , Y. Han , P. Hao , X. S. Liu , Br. J. Haematol. 2021, 195, 267. 34409610 10.1111/bjh.17775

[16] H. Tan , H. Liao , L. Zhao , Y. Lu , S. Jiang , D. Tao , Y. Liu , Y. Ma , Sci. Rep. 2017, 7, 46376. 28393858 10.1038/srep46376 PMC5385498

[17] W. Gan , X. Dai , X. Dai , J. Xie , S. Yin , J. Zhu , C. Wang , Y. Liu , J. Guo , M. Wang , J. Liu , J. Hu , R. J. Quinton , N. J. Ganem , P. Liu , J. M. Asara , P. P. Pandolfi , Y. Yang , Z. He , G. Gao , W. Wei , Nat. Cell Biol. 2020, 22, 246. 32015438 10.1038/s41556-020-0463-6 PMC7076906

[18] G. Bertolin , R. Ferrando‐Miguel , M. Jacoupy , S. Traver , K. Grenier , A. W. Greene , A. Dauphin , F. Waharte , A. Bayot , J. Salamero , A. Lombes , A. L. Bulteau , E. A. Fon , A. Brice , O. Corti , Autophagy 2013, 9, 1801. 24149440 10.4161/auto.25884

[19] M. T. Zilber , S. Gregory , R. Mallone , S. Deaglio , F. Malavasi , D. Charron , C. Gelin , Proc. Natl. Acad. Sci. U S A 2000, 97, 2840. 10706632 10.1073/pnas.050583197 PMC16017

[20] a) H. F. Guo , C. W. Vander Kooi , J. Biol. Chem. 2015, 290, 29120; 26451046 10.1074/jbc.R115.687327 PMC4705917

[21] E. Proietti , S. Rossini , U. Grohmann , G. Mondanelli , Trends Immunol. 2020, 41, 1037. 33055013 10.1016/j.it.2020.09.007

[22] H. Kolb , K. Kempf , M. Rohling , M. Lenzen‐Schulte , N. C. Schloot , S. Martin , BMC Med. 2021, 19, 313. 34879839 10.1186/s12916-021-02185-0 PMC8656040

[23] Y. Osawa , H. Kanamori , E. Seki , M. Hoshi , H. Ohtaki , Y. Yasuda , H. Ito , A. Suetsugu , M. Nagaki , H. Moriwaki , K. Saito , M. Seishima , J. Biol. Chem. 2011, 286, 34800. 21841000 10.1074/jbc.M111.235473 PMC3186417

[24] X. Huang , B. Hussain , J. Chang , CNS Neurosci. Therapeu. 2021, 27, 36. 10.1111/cns.13569 PMC7804893 33381913

[25] A. L. Page , G. Dupuis , E. H. Frost , A. Larbi , G. Pawelec , J. M. Witkowski , T. Fulop , Exp. Gerontol. 2018, 107, 59. 29275160 10.1016/j.exger.2017.12.019

[26] a) L. B. Jaeger , S. Dohgu , R. Sultana , J. L. Lynch , J. B. Owen , M. A. Erickson , G. N. Shah , T. O. Price , M. A. Fleegal‐Demotta , D. A. Butterfield , W. A. Banks , Brain, Behav., Immun. 2009, 23, 507; 19486646 10.1016/j.bbi.2009.01.017 PMC2783557

[27] S. Takeda , N. Sato , K. Ikimura , H. Nishino , H. Rakugi , R. Morishita , Neurobiol. Aging 2013, 34, 2064. 23561508 10.1016/j.neurobiolaging.2013.02.010

[28] a) K. Chandrasekaran , K. Hatanpaa , S. I. Rapoport , D. R. Brady , Brain Res. Mol. Brain Res. 1997, 44, 99; 9030703 10.1016/s0169-328x(96)00191-x

[29] A. Ronit , J. Haissman , D. M. Kirkegaard‐Klitbo , T. S. Kristensen , A. M. Lebech , T. Benfield , J. Gerstoft , H. Ullum , L. Kober , A. Kjaer , K. Kofoed , J. Vestbo , B. Nordestgaard , J. Lundgren , S. D. Nielsen , BMC Infect. Dis. 2016, 16, 713. 27887644 10.1186/s12879-016-2026-9 PMC5124288

[30] a) A. T. Ambikan , H. Yang , S. Krishnan , S. Svensson Akusjarvi , S. Gupta , M. Lourda , M. Sperk , M. Arif , C. Zhang , H. Nordqvist , S. M. Ponnan , A. Sonnerborg , C. J. Treutiger , L. O'Mahony , A. Mardinoglu , R. Benfeitas , U. Neogi , Cell Syst. 2022, 13, 665; 35933992 10.1016/j.cels.2022.06.006 PMC9263811

[31] B. Wang , A. M. Mezlini , F. Demir , M. Fiume , Z. Tu , M. Brudno , B. Haibe‐Kains , A. Goldenberg , Nat. Methods 2014, 11, 333. 24464287 10.1038/nmeth.2810

[32] S. Pai , S. Hui , R. Isserlin , M. A. Shah , H. Kaka , G. D. Bader , Mol. Syst. Biol. 2019, 15, e8497. 30872331 10.15252/msb.20188497 PMC6423721

[33] R. Carapito , R. Li , J. Helms , C. Carapito , S. Gujja , V. Rolli , R. Guimaraes , J. Malagon‐Lopez , P. Spinnhirny , A. Lederle , R. Mohseninia , A. Hirschler , L. Muller , P. Bastard , A. Gervais , Q. Zhang , F. Danion , Y. Ruch , M. Schenck , O. Collange , T. N. Chamaraux‐Tran , A. Molitor , A. Pichot , A. Bernard , O. Tahar , S. Bibi‐Triki , H. Wu , N. Paul , S. Mayeur , et al., Sci. Transl. Med. 2022, 14, eabj7521. 34698500 10.1126/scitranslmed.abj7521

[34] G. Csardi , T. Nepusz , Inter J., Comp. Syst. 2006, 1695, 1.

[35] V. D. Blondel , J.‐L. Guillaume , R. Lambiotte , E. Lefebvre , J. Stat. Mech.: Theory Exp. 2008, 2008, P10008.

[36] A. M. Newman , C. B. Steen , C. L. Liu , A. J. Gentles , A. A. Chaudhuri , F. Scherer , M. S. Khodadoust , M. S. Esfahani , B. A. Luca , D. Steiner , M. Diehn , A. A. Alizadeh , Nat. Biotechnol. 2019, 37, 773. 31061481 10.1038/s41587-019-0114-2 PMC6610714

[37] H. Wickham , 2016, ISBN 978‐3‐319‐24277‐4.

[38] D. Otasek , J. H. Morris , J. Bouças , A. R. Pico , B. Demchak , Genome Biol. 2019, 20, 185. 31477170 10.1186/s13059-019-1758-4 PMC6717989

[39] M. I. Love , W. Huber , S. Anders , Genome Biol. 2014, 15, 550. 25516281 10.1186/s13059-014-0550-8 PMC4302049

