# Supplementary material for: Host Plasma Microenvironment in Immunometabolically Impaired HIV Infection Leads to Dysregulated Monocyte Function and Synaptic Transmission Ex Vivo
Source: Adv Sci (Weinh). 2025 Feb 27;12(16):2416453. doi: 10.1002/advs.202416453 (PMC12021100; doi:10.1002/advs.202416453)
Supplement: Supplementary file 1 — Supporting Information [file ADVS-12-2416453-s002.pdf]

ADVANCED SCIENCE
Open Access

Supporting Information

for *Adv. Sci.*, DOI 10.1002/advs.202416453

Host Plasma Microenvironment in Immunometabolically Impaired HIV Infection Leads to Dysregulated Monocyte Function and Synaptic Transmission *Ex Vivo*

*Flora Mikaeloff, Marco Gelpi, Alejandra Escós, Tianqi Wang, Soham Gupta, Anna Olofsson, Sara Svensson Akusjärvi, Sabrina Schuster, Prajakta Naval, Vikas Sood, Negin Nikouyan, Andreas D. Knudsen, Beate Vestad, Julie Høgh, Johannes R. Hov, Thomas Benfield, Marius Trøseid, Vinay Pawar, Marijana Rucevic, Rui Benfeitas, Ákos Végvári, Liam O'Mahony, Rajkumar Savai, Niklas K. Björkström, Magda Lourda, João Pedro de Magalhães, Siegfried Weiss, Adil Mardinoglu, Mukesh Kumar Varshney, Annika C. Karlsson, Yasir Ahmed Syed, Susanne D. Nielsen and Ujjwal Neogi**

Supplementary Materials

Host plasma microenvironment in immunometabolically impaired HIV infection leads to dysregulated monocyte function and synaptic transmission *ex vivo*

Flora Mikaeloff[1#], Marco Gelpi[2#], Alejandra Escós[1], Tianqi Wang[3], Soham Gupta[1], Anna Olofsson[4], Sara Svensson Akusjärvi[1], Sabrina Schuster[1], Prajakta Naval[5], Vikas Sood[1], Negin Nikouyan[1], Andreas D. Knudsen[2], Beate Vestad[6,7], Julie Høgh[2], Johannes R. Hov[4,7,8], Thomas Benfield[9], Marius Trøseid[6,10], Vinay Pawar[1,11], Marijana Rucevic[11], Rui Benfeitas[1,12], Ákos Végvári[5], Liam O'Mahony[13], Rajkumar Savai[14,15], Niklas K. Björkström[16], Magda Lourda[16,17], João Pedro de Magalhães[18], Siegfried Weiss[19], Adil Mardinoglu[20,21], Mukesh Kumar Varshney[22], Annika C. Karlsson[4], Yasir Ahmed Syed[3], Susanne D. Nielsen[2], Ujjwal Neogi[1,*]

[1]The Systems Virology Lab, Division of Clinical Microbiology, Department of Laboratory Medicine, Karolinska Institutet, Stockholm, Sweden.
[2]Copenhagen University Hospital Rigshospitalet, Copenhagen, Denmark
[3]Neuroscience and Mental Health Innovation Institute and School of Bioasceinces, Hadyn Ellis Building, Cardiff University, Cardiff, United Kingdom.
[4]Division of Clinical Microbiology, Department of Laboratory Medicine, Karolinska Institutet, Stockholm, Sweden
[5]Division of Chemistry I, Department of Medical Biochemistry and Biophysics, Karolinska Institutet, Stockholm, Sweden
[6]Research Institute of Internal Medicine, Oslo University Hospital Rikshospitalet, Oslo, Norway
[7]Norwegian PSC Research Center, Oslo University Hospital Rikshospitalet, Oslo, Norway
[8]Institute of Clinical Medicine, University of Oslo, Oslo, Norway
[9]Department of Infectious Diseases, Copenhagen University Hospital – Amager and Hvidovre, 19 Hvidovre, Denmark
[10]Institute of Clinical Medicine, Oslo, Norway
[11]Olink AB, Uppsala, Sweden
[12]National Bioinformatics Infrastructure Sweden (NBIS), Science for Life Laboratory, Department of Biochemistry and Biophysics, Stockholm University, SE-106 91 Stockholm, Sweden
[13]Departments of Medicine and Microbiology, APC Microbiome Ireland, University College Cork, Cork, Ireland
[14]Lung Microenvironmental Niche in Cancerogenesis, Institute for Lung Health (ILH), Justus Liebig University, Giessen, Germany
[15]Department of Lung Development and Remodeling, Max Planck Institute for Heart and Lung Research, member of the German Center for Lung Research (DZL), member of the Cardio-Pulmonary Institute (CPI), Bad Nauheim, Germany
[16]Center for Infectious Medicine, Department of Medicine Huddinge, Karolinska Institutet, Karolinska University Hospital, Stockholm, Sweden
[17]Childhood Cancer Research Unit, Department of Women's and Children's Health, Karolinska Institutet
[18]Genomics of Ageing and Rejuvenation Lab, Institute of Inflammation and Ageing, University of Birmingham, B15 2WB, United Kingdom
[19]Department of Molecular Immunology, Helmholtz Centre for Infection Research, Braunschweig, Germany
[20]Science for Life Laboratory, KTH - Royal Institute of Technology, Stockholm, Sweden
[21]Centre for Host-Microbiome Interactions, Faculty of Dentistry, Oral & Craniofacial Sciences, King's College London, London, United Kingdom
[22]Department of Biosciences and Nutrition, Karolinska Institutet, Huddinge, Sweden

[#]Equal contribution
*Corresponding author. Email: ujjwal.neogi@ki.se

Supplementary Methods

Plasma secretome analysis: We used Olink® Explore, a multiplex immunoassay protein biomarker platform that detects ~3000 proteins in plasma. The platform uses Proximity Extension Assay (PEA) technology run on Illumina NovaSeq 6000 system (Illumina, US). The list of proteins is given in Table S15.

Sample Preparation. The 3k Explore protocol starts with sample preparation, where each sample is split into dilutions, corresponding to one or more blocks throughout the eight Explore 3k panels. Samples as well as the control samples are serially diluted using Olink Sample Dilution buffer, giving rise to five different dilutions: undiluted sample, 1:10, 1:100, 1:1000 and 1:100 000 dilution. The 1:100 000 dilution is a new addition to the sample preparation protocol for Explore 3k, since the 3k iteration of Explore contains a collection of high abundant biomarkers, that require this extra dilution step to ensure that they remain withing the range of their related assay. All dilutions are performed with the help of the Dragonfly Discovery and Mosquito LV liquid handling instruments from SPT Labtech, automating large parts of the workflow as well as allowing for reduction of sample and reagent consumption. Because of this, Explore 3k only requires a total volume of 20 µL from each test-sample to run all eight panels.

Sample Incubation. The Incubation mixes, 32 in total, for each block and panel are set up and combined with the samples of appropriate dilution. In this way each sample will be combined with each block ensuring that the samples are tested across all assays yet ensuring that those assays are within appropriate range and will be amplified properly in the first PCR step (PCR1). The samples are incubated at +4°C overnight. The incubation setup is performed by the Mosquito LV liquid handling instruments used in previous steps. Mirroring the original Explore 1.5k protocol, in Explore 3k the Mosquito instrument combines 0.6 µL Incubation mix with 0.2 µL sample ensuring that a minimal amount of plasma or serum sample is consumed for the Explore run.

PCR1 and PCR2. After incubation PCR1 mix is added to all reactions by the Dragonfly Discovery and PCR1 is performed. This step extends and amplifies the DNA target sequence arising from the probe-antigen complex that has formed during the incubation step. After PCR1, the four blocks in each panel are pooled according to sample in a PCR1 pooling plate. For Explore 3k this will result in two 384-well pooling plates, where the original Explore panels are collected in the first plate and the new Explore panels are collected in the second. In practical terms, post-PCR1, the Explore 1.5k workflow is duplicated for the new panels in Explore 3k.

Material from the PCR1 pooling plates is used as template when setting up a new PCR, PCR2, where the pooled PCR1 template, PCR2 master mix and sample indexes are combined. The individual indexes are disseminated so that each sample across all eight panels will be represented by the same index. The index is incorporated into the PCR2 product during amplification. Once PCR2 is completed the PCR2 reactions are pooled panel wise to generate eight library tubes. These steps in the workflow are automated and performed by the Eppendorf's epMotion 5075lc in the standard Olink protocol.

Bead purification and Library quality control. The eight libraries are bead-purified using the AMPure XP magnetic beads purification protocol (Beckman Coulter), removing primers and other DNA fragments that might impede correct NGS readout. Libraries are run on Agilent's 2100 Bioanalyzer using the Agilent High Sensitivity DNA kit for the purpose of quality control.

Next generation sequening. The Explore libraries are diluted to the proper concentration according to Olink specification and prepped using the Illumina NovaSeq Xp workflow, enabling the libraries for each panel to be run on separate lanes on the flowcells. Standard procedure at Olink is to run libraries on the Illumina NovaSeq 6000 system. In Explore 3k, custom recipes are used for the NovaSeq 6000 that utilizes dark cycles for common regions. Hence no PhiX needs to be added to the libraries before sequencing, leading to increased library reads. The sequence data is processed and normalized to produce Olinks relative quantification unit Normalized Protein eXpression (NPX)(*1*). Briefly, counts of matched sequence reads for each combination of assay and sample barcodes are divided by the counts of extension controls (ExtCtrl) with the same sample barcode. These controls are spiked into every sample at known concentrations in the immunoreaction step. For assay i in sample j, the non-normalized NPX (ExtNPX) is thus defined as:

ExtNPXi,j=log2 (Counts(SamplejAssayi)Counts(ExtCtrlj))ExtNPXi,j=log2 CountsSamplejAssayiCountsExtCtrlj

The plate control sample, consisting of pooled plasma run in triplicate on each plate, is used to correct for variation between plates. This is done by subtracting the median of the plate control ExtNPX per assay and plate:

NPXi,j,k=ExtNPXi,j,k−median(ExtNPXplateCtrls i,k)NPXi,j,k=ExtNPXi,j,k−medianExtNPXplateCtrls i,k

where k = plate. To further minimize potential technical variation across plates, an additional normalization step can be performed by subtracting the median NPX value per assay and plate:

NPXIntNorm i,j,k=NPXi,j,k−median(NPXi,k)NPXIntNorm i,j,k=NPXi,j,k−medianNPXi,k

This step is referred to as intensity normalization and is only recommended when samples are adequately randomized over plates.

The additional internal and external controls included in every run are used to monitor technical consistency and quality according to Olinks standard criteria (*1*).

References:

1. L. Wik *et al.*, Proximity Extension Assay in Combination with Next-Generation Sequencing for High-throughput Proteome-wide Analysis. *Mol Cell Proteomics* **20**, 100168 (2021).

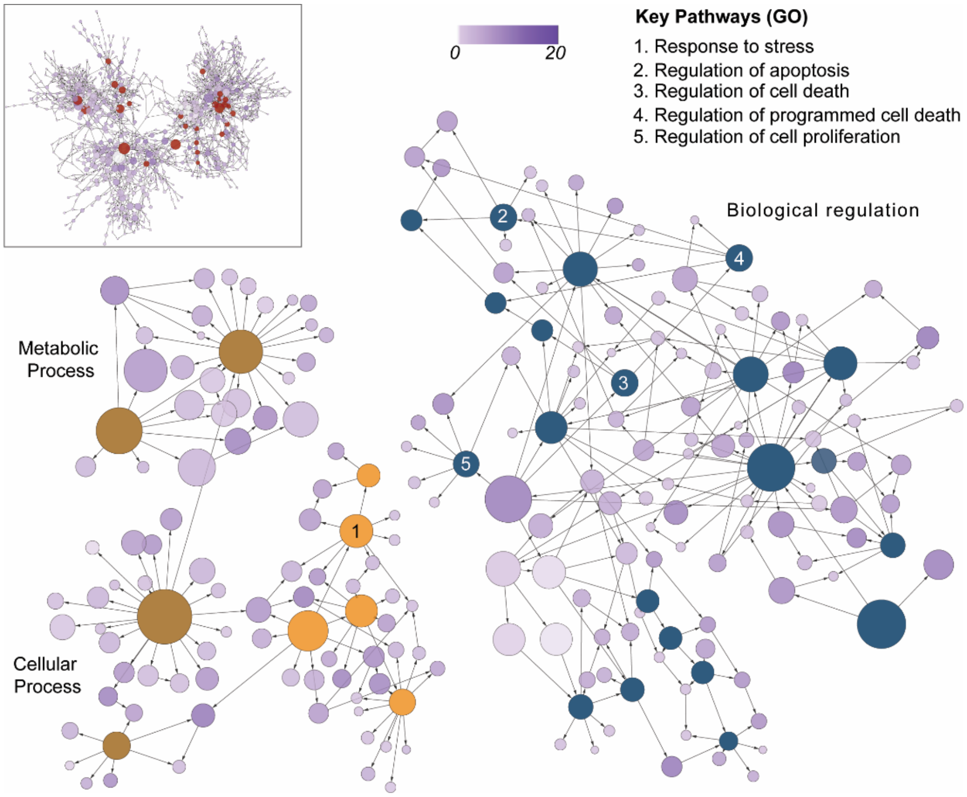

Fig S1. Cytoscape network with BINGO plugin to find enriched Gene Ontology: Biological Processes among proteins differing HC-like and at-risk clusters. The node size is proportional to the number of proteins annotated with this process, and color varies based on p-value. The top 30 pathways are colored red in the general network (up). The top 30 pathways and first neighbors are zoomed in (bottom).

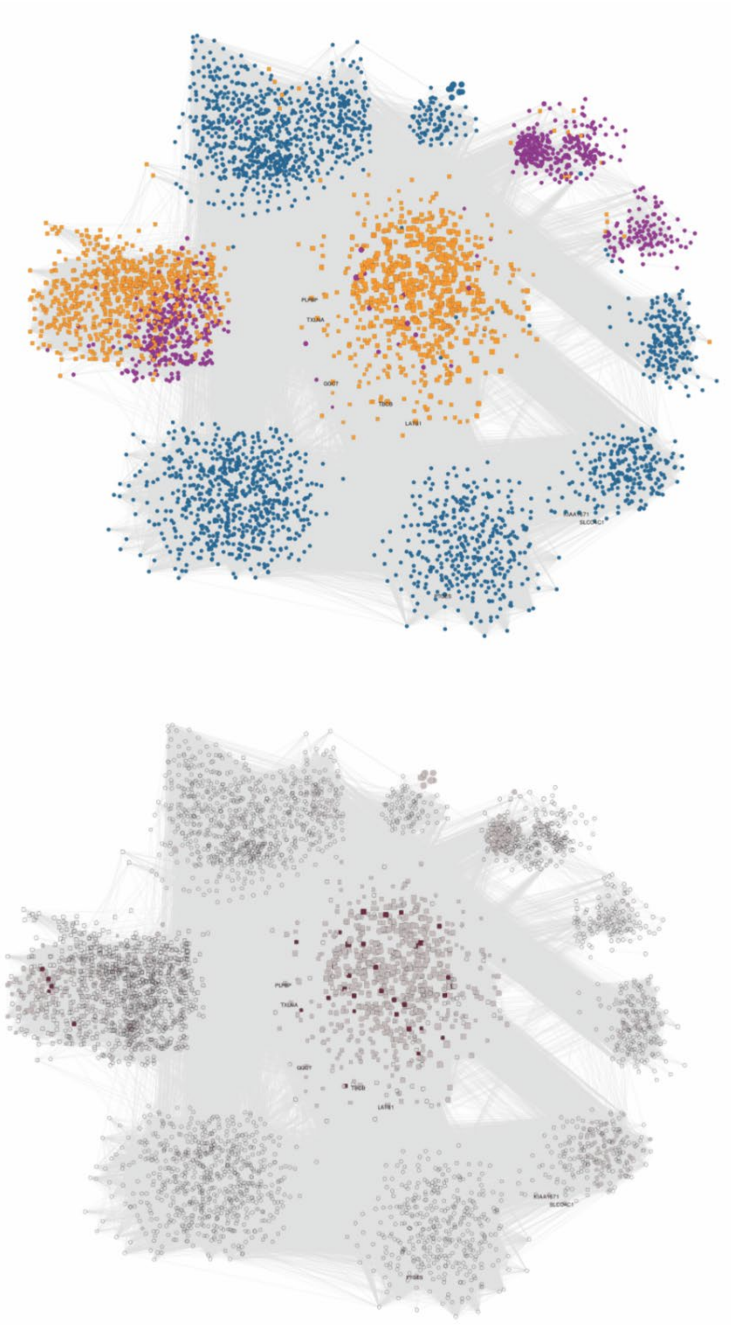

Fig S2. Co-expression network after communities detection based on transcriptomics, metabolomics and proteomics. Node color is determined by type of feature (metabolites = purple, proteins = orange, genes = blue) or SASP proteins (significant = red, non-significant = grey, non SASP = transparent) (top). Driver genes identified by structural causal modelling are labelled.

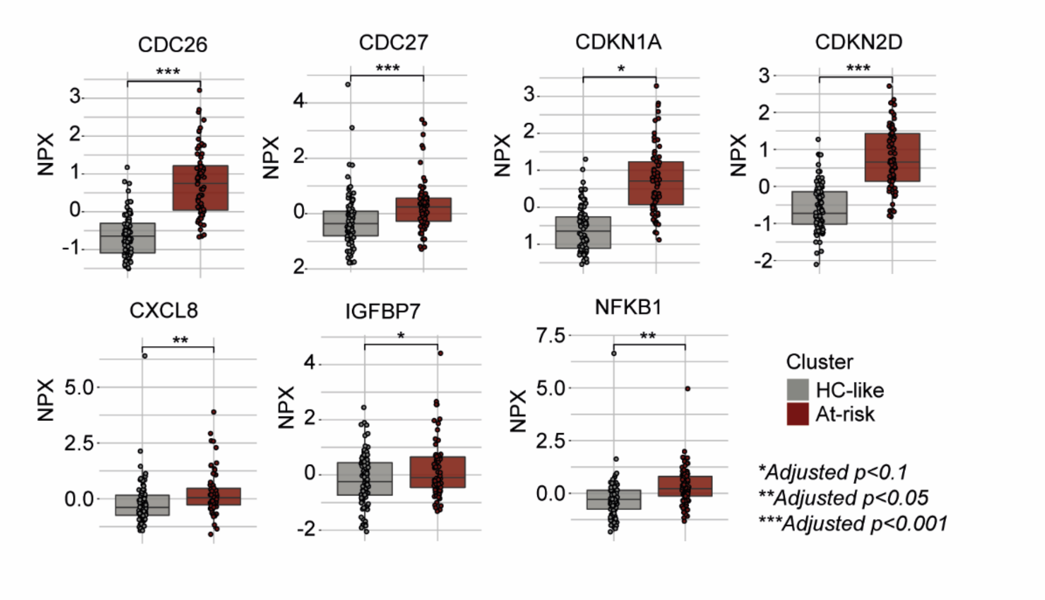

Fig S3. Level of SASP proteins.

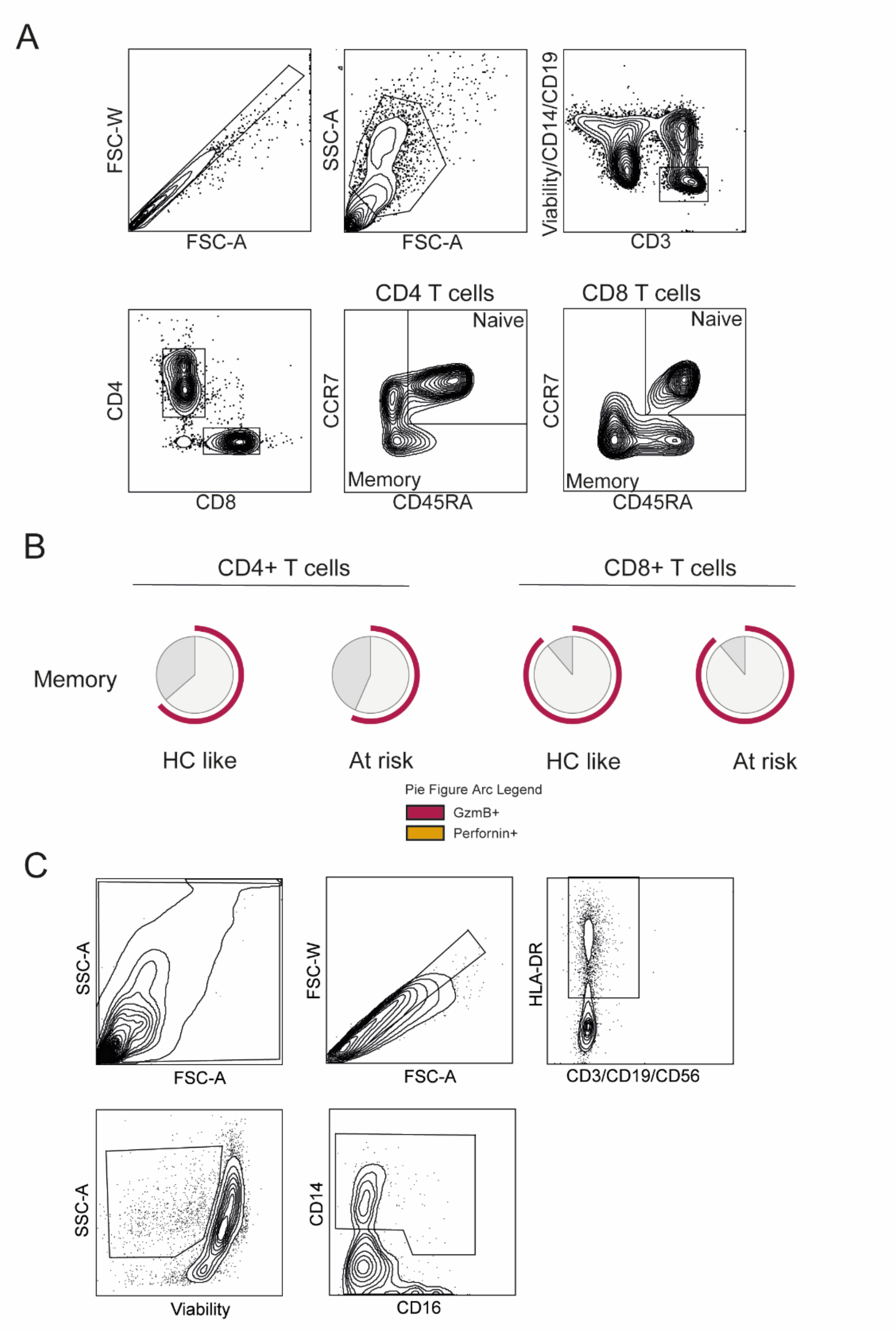

Fig S4. Functional analysis of T cells and monocytes. A) Plots show the gating strategy for live, memory $CD4^{+}$ and CD8 T^{+} cells analyzed using flow cytometry after a 48h stimulation. Naïve $CD4^{+}$ and $CD8^{+}$ T cells were excluded from further analysis. B) Pie charts with arcs depicting the co-expression of Granzyme B (GzmB) and Perforin on memory $CD4^{+}$ and CD8 T^{+} cells after incubation with HC-like or at-risk plasma. Charts were generated using SPICE. C) Plots show the gating strategy for live monocytes, grouped together classical $CD14^{+}$, intermediate $CD14^{+}$/$CD16^{+}$ and non-classical $CD16^{+}$, analyzed using flow cytometry after a 48h stimulation.

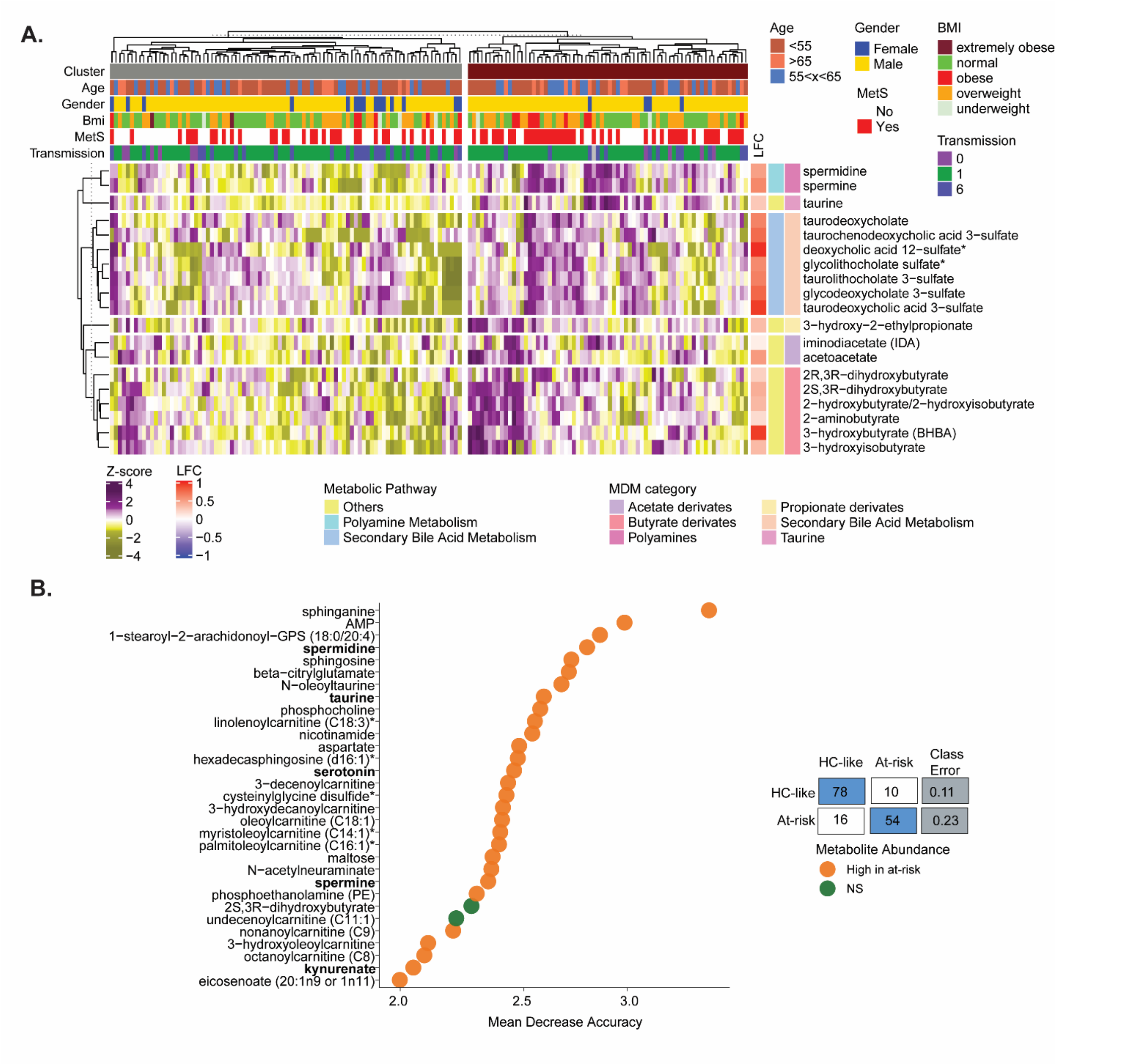

Fig S5.. A) Heatmap of microbiome-associated metabolites significantly differing between HC-like and at-risk. Data were z-score transformed. LogFC between HC-like and at-risk, category, and pathway are presented for each metabolite. For each patient, age, gender, BMI, and transmission mode is indicated. B) Box plot showing the difference in the metabolites of glutamate metabolism and neurosteroids between the HC-like and at-risk groups. C) Random forest analysis to identify the top metabolites.

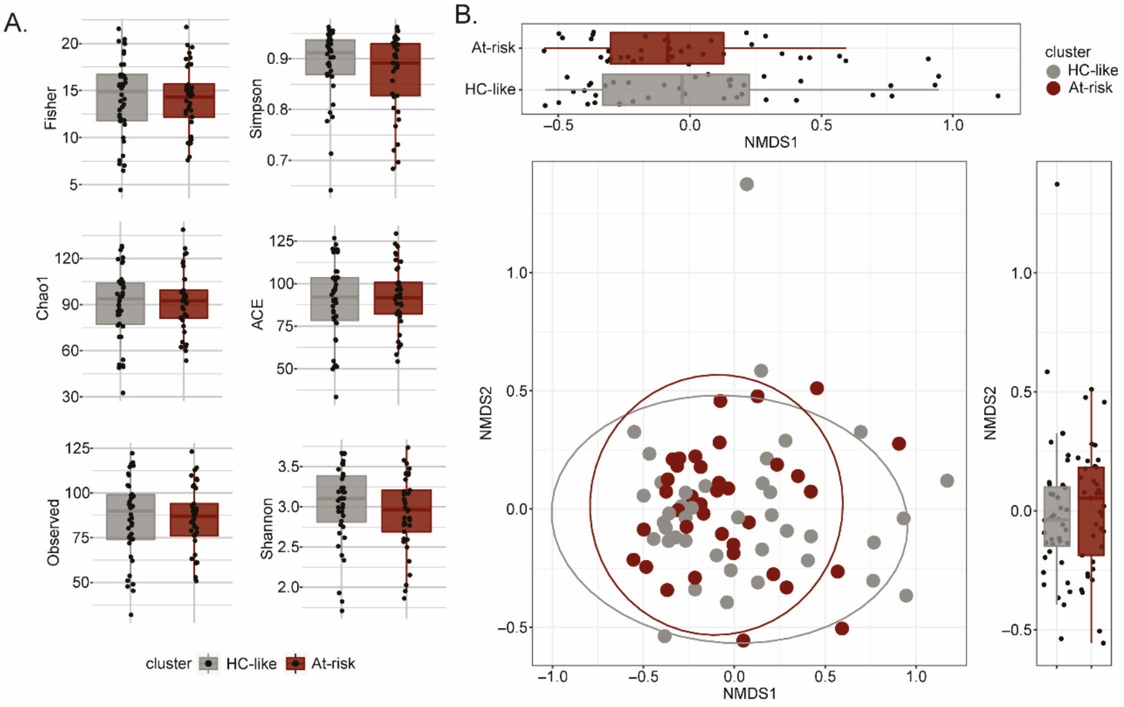

Fig S6:(A) Boxplot of alpha diversity indexes and (B) non-metric multidimensional scaling (NMDS) plot of Bray- Curtis distances (beta diversity) of HC-like and at-risk groups. Boxplots based on NMDS1 and NMDS2 values are indicated

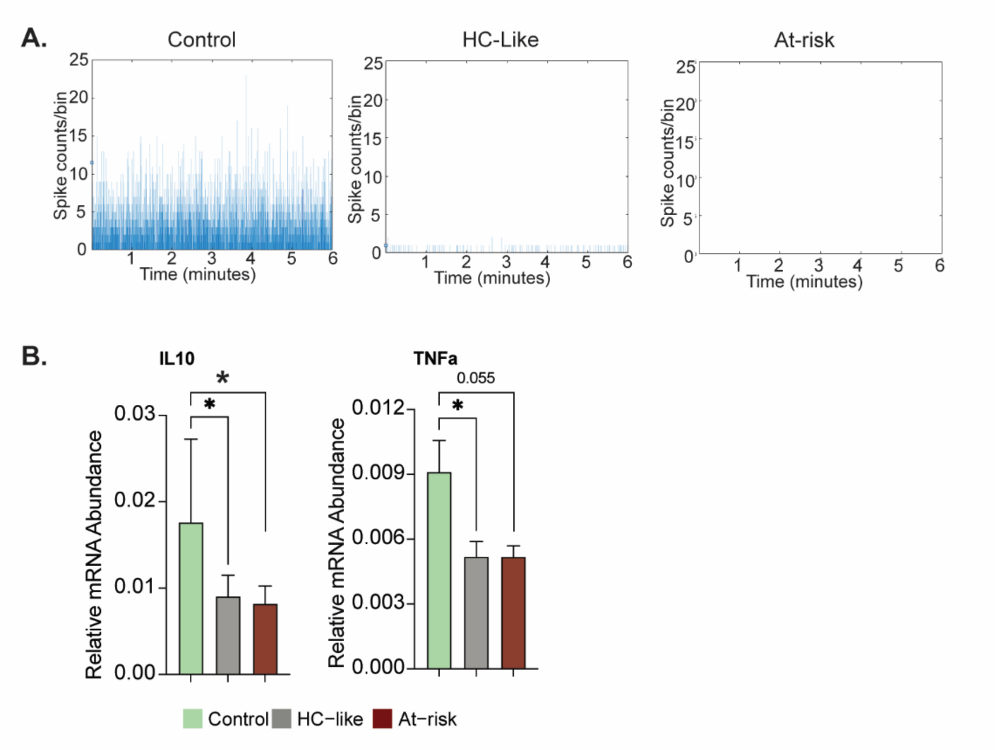

Fig S7. Cortical forebrain organoids from induced pluripotent stem cells (iPSC) display reduced electrical network activity and altered inflammatory response when exposed to plasma from HC-like and or an at-risk group. A) Representative Array-Wide Spike Detection Rate (ASDR) plots from iPSC-derived organoids from control, HC-like, and at-risk groups, when 2-month-old brain organoids were incubated with 20% plasma from HC-Like and at-risk for 3 days. B) Bar graph showing the expression level of IL-10 and TNF-α from control and plasma-treated organoids (n=3). Data sets were analyzed with one-way ANOVA with post hoc comparisons using Dunnett's multiple comparisons test compared to control samples. Stars above points represent Dunnett-corrected post hoc tests. All data are presented as means ± SEM *P<0.05; **P<0.01; ***P<0.001 ****P<0.0001 vs. control.

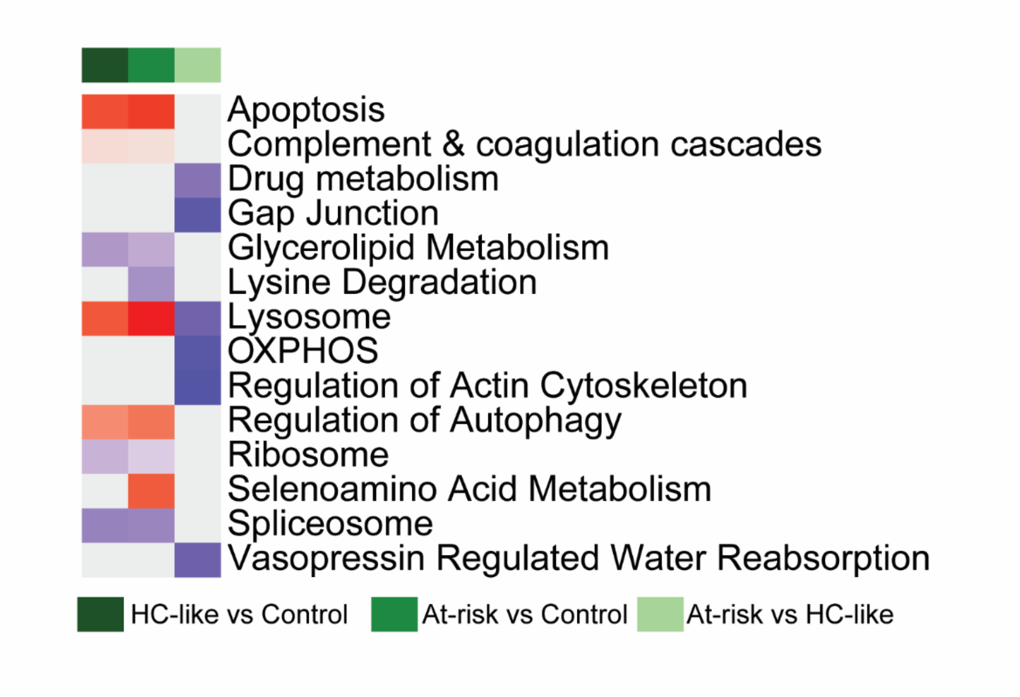

Fig S8. Protein set enrichment analysis in different groups.

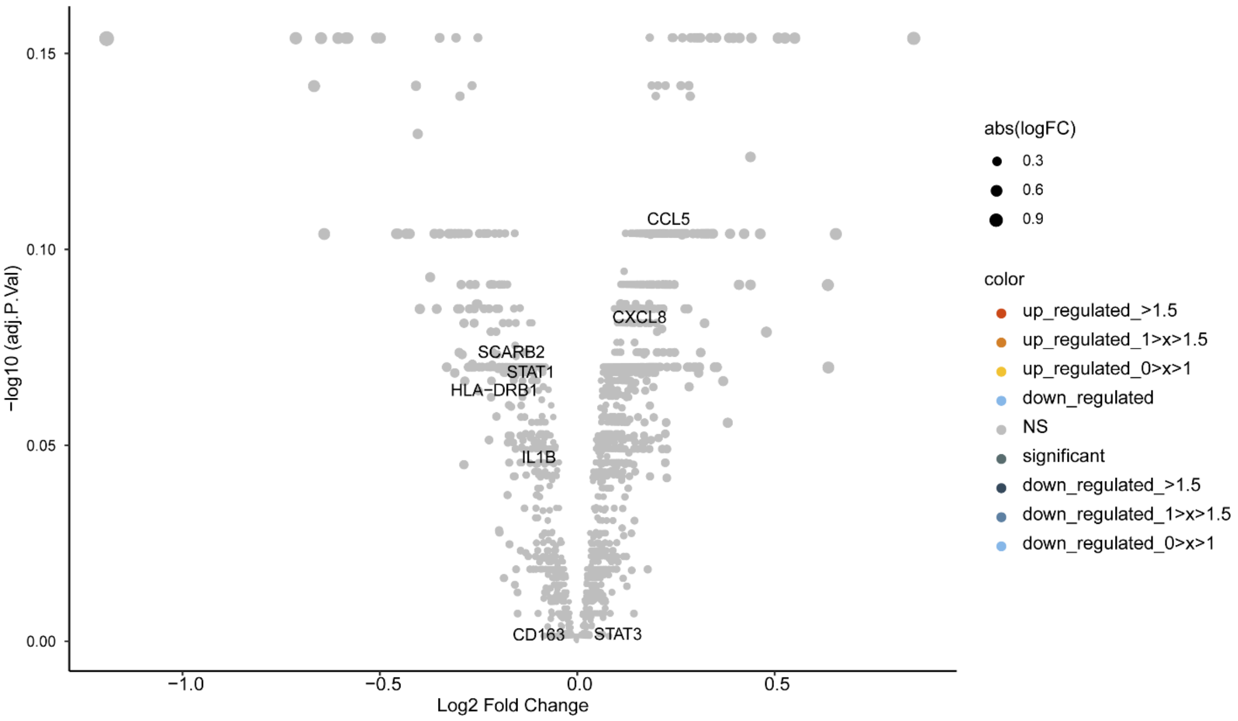

Fig S9. Volcano plots show the proteins differing between spermine-treated cells and untreated cells.
